# MIFE and MIFD: Minimum information for fermentation experiments and devices

**DOI:** 10.1093/gigascience/giag038

**Published:** 2026-04-09

**Authors:** Georgios K Georgakilas, Brett Metcalfe, Ariane Bize, Matthew Crowther, Emilie Fernandez, Susana Maria Alonso Villela, Stuart Owen, Rudolf Wittner, David Camilo Corrales, Anselm von Gladiss, Peter Blomberg, Munazah Andrabi, Cesar Arturo Aceves Lara, Hans Mattila, Marily Wiebe, Theodore Dalamagas, Jasper J Koehorst

**Affiliations:** Information Management Systems Institute, ATHENA Research Center, 15125 Marousi, Greece; Department of Earth Sciences, Faculty of Science, Vrije Universiteit Amsterdam, 1081 HV, Amsterdam, The Netherlands; Amsterdam University College, 1098 XG, Amsterdam, The Netherlands; Université Paris-Saclay, INRAE, PROSE, 92160 Antony, France; School of Computing Science, Newcastle University, NE1 7RU Newcastle Upon Tyne, United Kingdom; INRAE, University of Montpellier, LBE, 11100 Narbonne, France; TBI, Université de Toulouse, CNRS, INRAE, INSA, 31077 Toulouse, France; Department of Computer Science, The University of Manchester, M13 9, PL, Manchester, United Kingdom; BBMRI-ERIC, Neue Stiftingtalstrasse 2/B/6, 8010 Graz, Austria; TBI, Université de Toulouse, CNRS, INRAE, INSA, 31077 Toulouse, France; Institute for Computer Science, University of Koblenz, 56070 Koblenz, Germany; VTT Technical Research Centre of Finland, 02044 VTT, Finland; Department of Computer Science, The University of Manchester, M13 9, PL, Manchester, United Kingdom; TBI, Université de Toulouse, CNRS, INRAE, INSA, 31077 Toulouse, France; VTT Technical Research Centre of Finland, 02044 VTT, Finland; VTT Technical Research Centre of Finland, 02044 VTT, Finland; Information Management Systems Institute, ATHENA Research Center, 15125 Marousi, Greece; UNLOCK, Wageningen University & Research and Delft University of Technology, The Netherlands

**Keywords:** metadata, biomanufacturing, industrial biotechnology, data management, metadata standardization, minimum information models, FAIR principles, data AI-readiness, fermentation experiments, fermentation devices

## Abstract

**Background:**

As the technological advancements of the early 21st century are pushing industrial biotechnology (IB) into the realm of Big Data–driven innovation, the requirement for trustworthy data management, annotation, and standardization is emerging as a necessity. Minimum information models (MIMs) have long been used across disciplines as the backbone of good data management practices by providing the scaffold upon which standardized recording of metadata can adequately and succinctly describe an understudied phenomenon.

**Findings:**

Here we present a minimum set of metadata, named the minimum information for fermentation experiments (MIFE) and devices (MIFD), that has been specifically designed to accommodate the data management and annotation needs of IB-related fermentation experiments. Although the proposed schema is tailored to IB applications, MIFE and MIFD build upon well-established models and community standards to facilitate easier integration with existing infrastructure and easier adoption by the community, as well as aim to integrate Findable, Accessible, Interoperable, and Reproducible (FAIR) principles in the IB field. In addition, the integration with FAIR Data Station (FAIR DS), a tool that offers metadata validation and enables the automated uptake of (meta)data from data management repositories such as FAIRDOM-SEEK, is showcased. The proposed models are accompanied by a Python package that enables their programmatic use by creating a Linked Data Modeling Language (LinkML) schema that can fuel subsequent analyses.

**Conclusions:**

Through the promotion and simplification of knowledge discovery, we believe that MIFE and MIFD can accelerate the application of state-of-the-art artificial intelligence (AI) methods and the adoption of explainable AI to better understand bioprocesses at scale.

## Background

The expectation of data sharing between and within institutions has begun to become the scientific norm in many disciplines of science [1]. Such endeavors are being further promoted by initiatives led by individuals, institutions, and funding and governmental agencies seeking to facilitate best practices through the adoption and promotion of the principles of both Findable, Accessible, Interoperable, and Reusable (FAIR) [2] and open science initiatives [3]. That is because good data management practices are not just a bureaucratic (or academic) exercise but are crucial for cultivating new knowledge, facilitating data sharing, and ensuring reproducibility [4, 5]. Such practices are even more essential for disciplines with tighter ties to the commercial domain, where regulatory agencies (e.g., for food, health care, drugs, vaccines, and pharmaceutical development) [6, 7] require that the provenance [8, 9] of the product is sufficiently well known. Industrial biotechnology (IB), bioprocess engineering [10], and synthetic biology are examples of one such group of disciplines. IB endeavors to incorporate biological know-how with engineering principles, to use (un)modified organisms grown under relatively restrictive conditions within a vessel to synthesize bio-based products and consumables or the building blocks toward them at an industrial scale [10].

### Minimum information models

As the adoption of good data management practices gathers pace, questions have now begun to switch from “why” to “what” metadata (i.e., data about data) should be required alongside datasets (e.g., [11–17]). One approach to ensure the recording of sufficient and relevant information is through the use of community-defined minimum information models (MIMs) [18, 19] that provide a set of guidelines and standardized metadata that are required to adequately describe an object or phenomenon under study [20]. The goal of minimum information about a microarray experiment (MIAME), for instance, was to ensure that there was a lack of ambiguity regarding both the interpretation and the reproduction of a microarray experiment [21]. MIMs allow a community to adopt specific criteria while giving a researcher the flexibility to describe, in detail, additional optional or recommended metadata if they so require. For example, specific metadata may fall into the categories of “must,” “should,” or “may (or optional)” [22] or “shall,” “if relevant,” “should” [23] when reporting. Here, we follow one convention of labeling the reporting requirement of metadata as “mandatory,” “recommended,” and “optional,” which can be defined as follows:

1. **Mandatory**. Aspects of metadata that the experimentalist is required to record and are crucial for understanding the data. For example, providing information about the microbial strain that was used in an experiment and the bioreactor conditions throughout a fermentation experiment, with the information of each sensor.
2. **Recommended**. Aspects of metadata that are encouraged to be cataloged but are not mandatory and can be treated flexibly. For example, providing an Open Researcher and Contributor Identifier (ORCID) for an author is recommended so as to unambiguously differentiate 2 or more John Smiths, but it is not strictly necessary as the same information can be gleaned elsewhere (e.g., institution and email).
3. **Optional**. Aspects of metadata that may be selected, thereby providing flexibility to add additional information that the user deems pertinent but is not strictly required to understand the dataset. For example, additional information may be provided about an organism under cultivation such as the initial isolation, potential synonyms, and extended taxonomic information.

The benefit of a MIM is that it allows focusing on the core information that a system’s behavior is based on. When analyzing systems, reducing the amount of information with a MIM means reducing the number of parameters to analyze. In such a model—through reducing the complexity—fundamental principles and key variables of a system can be identified better. Thus, MIMs help in analyzing systems and testing hypotheses. In addition, MIMs not only facilitate data sharing and system analysis but are also a big step toward standardized and consistent documentation, and they further motivate standardized experimental settings. Examples of such standardization initiatives include MIAME [24] and the minimum information requested in the annotation of biochemical models (MIRIAM) [25]. This claim holds especially for IB, as MIMs ensure consistent metadata and thus comparability of experimental results. In complex settings such as bioprocesses, clear guidelines on metadata collection reduce the number of missteps taken in development. Together with an improvement in experimental consistency, this may help in replicating and scaling up processes, translating research and development into commercial production, and facilitating regulatory approvals.

### Minimum information models in industrial biotechnology

**Figure 1:**
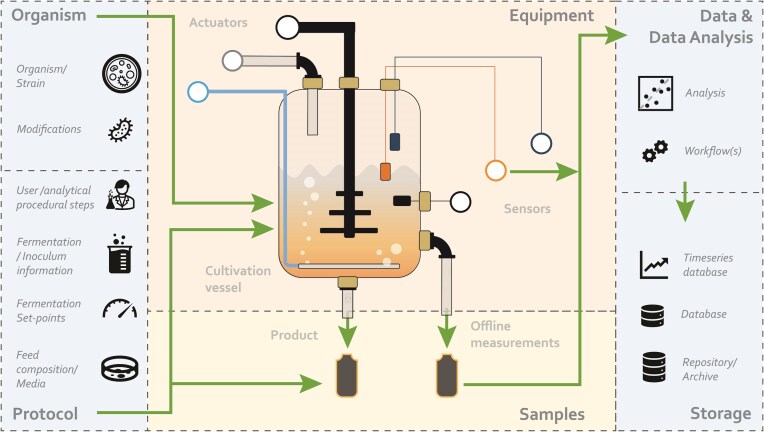
Information necessary to understand the data associated with an industrial biotechnology fermentation experiment. Green arrows show the relationships between the various aspects of information.

To make sense of IB (experimental) data and ensure that users of such data understand what will occur, is occurring, and has occurred (Fig. 1) necessitates that a data consumer should at least be provided with (i) information on the organism, any modifications, or particular strains; (ii) information on the cultivation equipment used, including type of sensors and actuators; (iii) a description and information on the experimental setup and protocol used, including set-points; (iv) information and a description of (any) sampling; and the (v) location and description of the data and data analysis. Furthermore, advancements in IB are predicted to allow for enhanced monitoring of bioprocesses through off-line, at-line, on-line, and in-line methods [26] and/or modeling of bioreactors via artificial intelligence (AI), machine learning (ML), and explainable AI (XAI) methods [27, 28]. Such advancements should enable more intuitive steering of the bioprocess—so-called Digital Twins [29]—but achieving this requires that data and metadata become standardized and machine readable.

A secondary consideration is to ensure traceability, which is handled by provenance, essentially information about entities, activities, and people involved in producing a piece of data or object [30]. As data and its precursors (e.g., biological or environmental samples) are typically shared between organizations, each of the organizations can provide information for only a part of the described object’s life cycle. In turn, this results in a situation where complete documentation of the object’s provenance is easily fragmented, as it is generated and can be managed by various heterogeneous institutions [31]. Such a scenario is becoming even more relevant in the context of the European strategy for data, namely, the Common European Data Spaces [32] or AI Act [33]. To enable full traceability of the dataset history, it is essential to enable linking the various, fragmented provenance pieces. Overcoming the challenges of fragmented provenance has been a focal point of provenance research in recent years [31] and is currently a subject of standardization within the ISO TC 276 Biotechnology WG 5 Data Processing and Integration, under the ISO 23494 Provenance Information Model for Biological Material and Data series [9]. This series provides a domain-agnostic integrative horizontal framework for provenance.

### Overview of existing knowledge organization systems

In contrast, MIMs are a crucial complement to such a provenance framework, as they add fundamental *domain-specific* information. Several existing MIMs and other types of knowledge organization systems (KOSs) are relevant to the field of IB and the fermentations it encompasses. However, to our knowledge, none of the currently available ones comprehensively cover the general and specific concepts needed to describe IB experiments. For example, the Sensor, Observation, Sample, and Actuator (SOSA) [34] as well as Semantic Sensor Network Ontology (SSN) [35] ontologies are dedicated to sensors, while PROV-O (the PROV ontology) [30] addresses provenance, and Friend-Of-A-Friend (FOAF) [36] focuses on describing persons and their relations. The Basic Formal Ontology (BFO) [37] developed by the Open Biological and Biomedical Ontology (OBO) Foundry is an upper-level ontology, helping to ensure consistency, interoperability, and logical coherence across diverse ontologies and knowledge domains.

The investigation, study, and assay (ISA) abstract model [38] and associated ISA tools are widely used in the field of biology. On this basis, an ontology has also been created, named Just Enough Results Model (JERM) [39]. While ISA represents a metadata framework for describing projects and associated experiments, it does not provide an adequate model for describing the equipment used in those projects, equipment that is central to IB fermentations. The ISA framework also lacks a conceptual hierarchical level that describes observation units [40] (e.g., a fermentation run), samples, fermentation devices, and sensors, among other items depicted in Fig. 1. One alternative is the Experimental Factor Ontology (EFO) [41], which provides a systematic description of many experimental variables related to molecular biology, but it also lacks many concepts specifically related to IB fermentation. For instance, it includes the concept of instrument, with 84 distinct specific instrument classes (e.g., centrifuge, spectrophotometer), but other key instruments for industrial biotechnology experiments are lacking, such as mass flow controllers and bioreactor vessels.

Regarding the description of processes, the Process and Observation Ontology (PO2) [42] and its associated management tool are especially relevant. It reuses concepts from upper-level and core ontologies (e.g., BFO, SOSA) and proposes generic concepts that can be specialized for domain ontologies. The concepts that can be specialized are component, step, attribute, process, material, method, and scale. PO2, however, lacks domain-specific terms for IB fermentation processes. The application core Ontology of Experimental Scientific Objects (OESO-CORE) [43] reuses concepts from core ontologies (e.g., FOAF, SOSA, PROV-O) and enables the description of concepts such as organization and experimental context, types of environment, equipment, and physical objects. Since it is not IB-oriented, it cannot fully support engineering and physical parameters critical to fermentation. The Environmental Biorefinery Ontology (EBO) [44] extends the core ontology PO2 and the OESO-CORE [43] to provide a framework for describing various aspects of environmental biorefinery processes. This includes the characterization of instruments, components, and processes involved in the transformation of biomass into valuable products. It has limitations in describing strains, their induction, and the conditions of a fermentation experiment.

Another alternative is the Environment Ontology (ENVO) [45] that is used to represent knowledge about environments, environmental processes, ecosystems, habitats, and related entities. However, being mostly related to the environment, most ENVO terms that could be relevant for IB, such as bioreactor, actually refer to environmental biotechnology processes (waste or wastewater treatment) rather than IB. Alongside the IB-adjacent KOS presented above, a significant number of additional KOS are relevant to IB experiments but restricted to specific aspects of IB. Nonexhaustive examples are Chemical Entities of Biological Interest (ChEBI) [46], Minimum Information about any (X) Sequence (MIxS) and its subcategories from the Genomics Standards Consortium [47], MIMs for other types of experimental data [24, 48, 49], and the BacDive repository [50] that is relevant to reference strains but does not yet include eukaryotes (e.g., yeast). Finally, the Ontology for Biomedical Investigations (OBI) [51] is a rich ontology that describes scientific and/or clinical investigations, the protocols they use as well as instrumentation and materials, the generated data, and the types of analysis performed upon the data. Although many individual concepts of OBI are relevant to the field of IB, its application to describe IB processes is limited because of its general orientation toward the biomedical field.

### Aims and objectives

**Figure 2:**
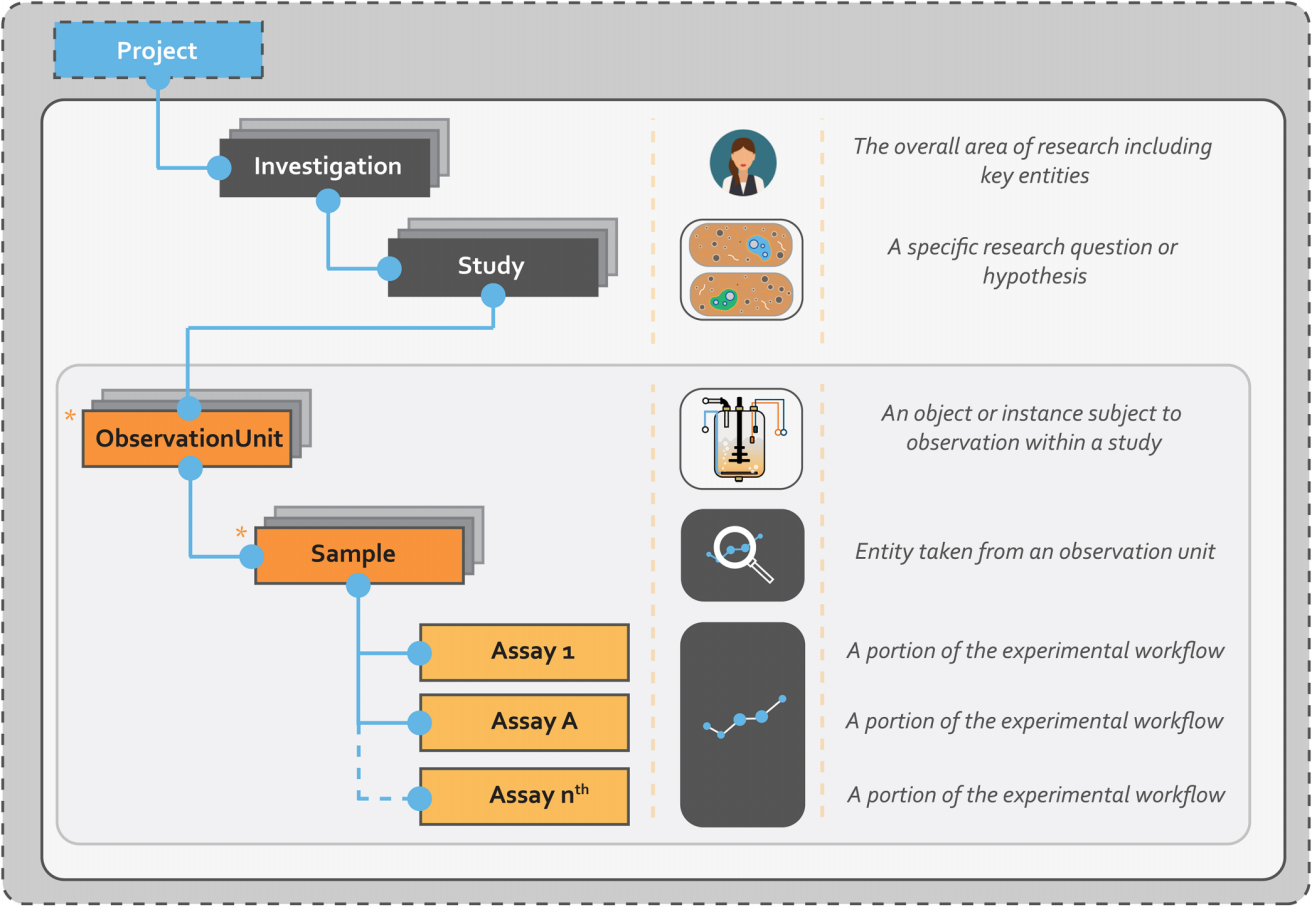
MIFE metadata hierarchy. MIFE uses an extended version of the ISA structure [38] by incorporating novel metadata terms related to the concepts of observation units and samples. The observation unit subset describes fermentation runs, while sample refers to entities taken from the culture at any step of the process. Each investigation may include many studies, and a study can be supported by multiple observation units from which culture volume entities can be taken to be processed with experimental assays. Levels with a asterisk represent the extension [40] to ISA [38].

**Figure 3:**
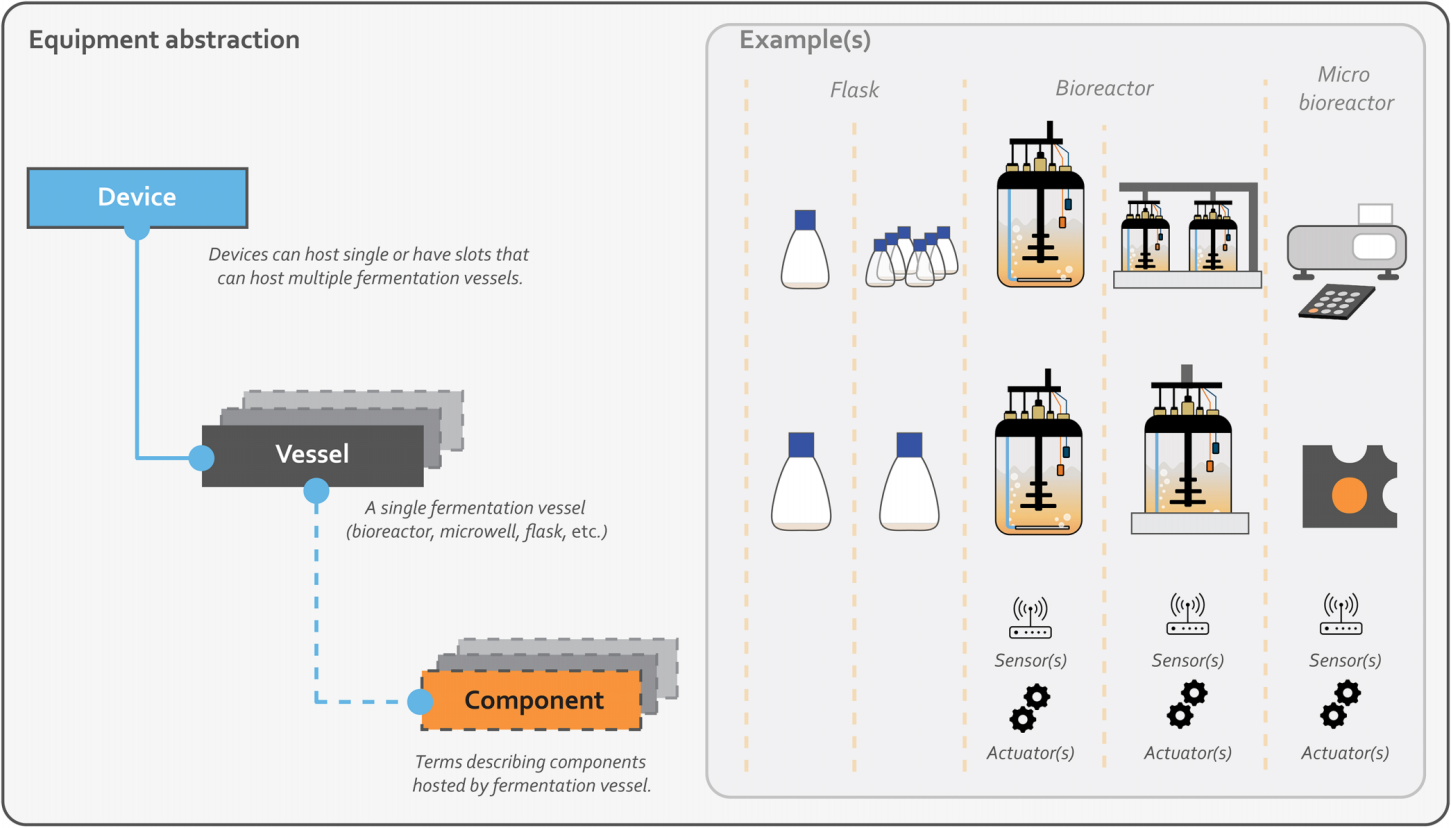
Overview of the MIFD metadata hierarchical structure. The proposed hierarchy ensures support for the majority of fermentation equipment configurations currently available in the IB field. Devices can contain a single (i.e., bioreactor) or multiple fermentation vessels (i.e., microwells), while each vessel can host multiple components (i.e., sensors and actuators).

Therefore, as there is no MIM specifically relevant for the needs of IB, here we introduce the minimum information for fermentation experiments (MIFE) and devices (MIFD) models that provide a set of standardized metadata schemas tailored to the field of IB (Figs. 2 and 3). These proposed models—available as ontologies deposited in BioPortal [52]—were designed as part of the wider efforts of the EU-funded project BioIndustry4.0 [53] (“RI Services to Promote Deep Digitalization of Industrial Biotechnology—Towards Smart Biomanufacturing”). To advance the technological landscape of bioindustry in the EU, BioIndustry4.0 combined interdisciplinary teams of computer scientists, bioinformaticians, biotechnologists, biologists, and chemical engineers, among others. MIFE and MIFD are the culmination of a 2-year effort to bring this community together through workshops and webinars, reaching a consensus on the design requirements of structured metadata related to fermentation experiments. The aim was to thoroughly describe IB experiments by adopting terms from IB-adjacent MIMs and ontologies found in the literature, supplemented with new terms that describe integral components of IB bioprocesses that are currently missing from existing models.

**Figure 4:**
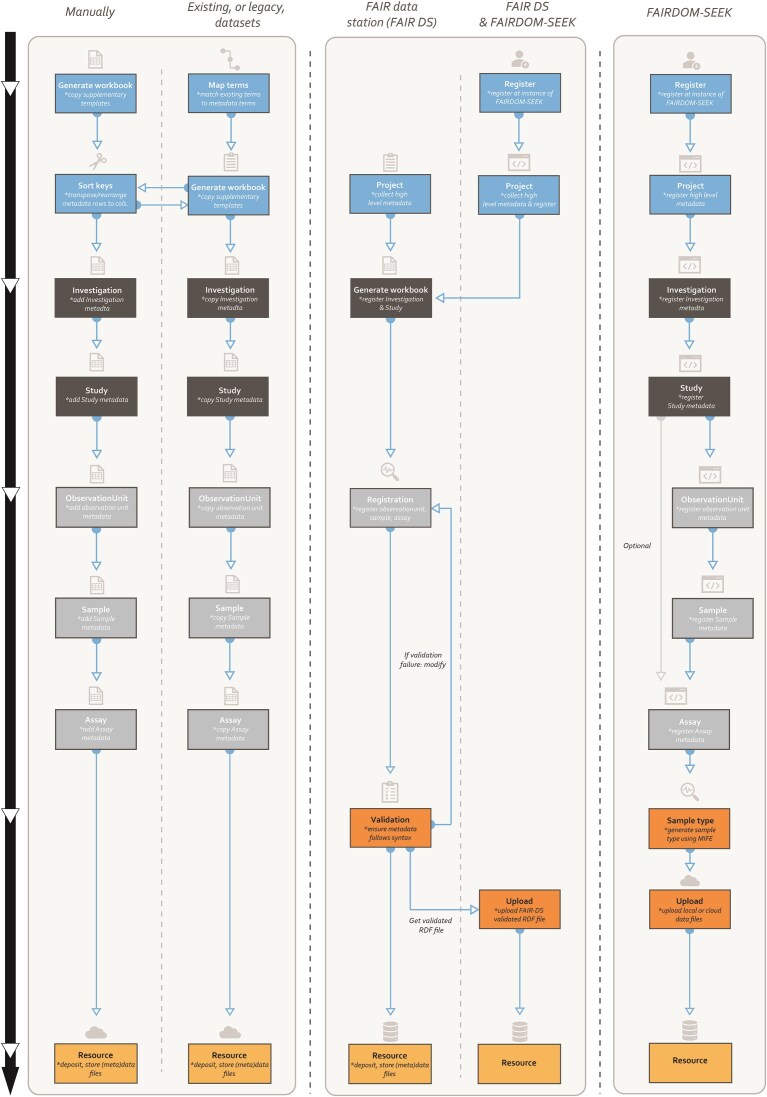
Flow diagram of how a user can use MIFE. An individual can choose to manually create their metadata file or to use FAIR DS, FAIRDOM-SEEK, or a combination of the two.

As a brief summary, MIFE was developed by using an extension of ISA [38] that also includes the concepts of observation units [40, 54] and samples that correspond to fermentation runs and samples taken from a fermentation vessel during or after a run is completed, respectively. The hierarchical nature of the extended ISA structure behind MIFE is capable of supporting the management of entire projects by enabling the thorough recording of key information related to fermentation experiments (Fig. 4). In addition to metadata regarding the purpose of the investigation or each experiment specifically and the people involved, MIFE also covers the operating conditions of a fermentation vessel, the strain used as a microbial cell factory, the samples taken throughout the process, and any assay applied on these samples (Fig. 2).

MIFE is complemented by MIFD, which is a hierarchical model for specifically capturing key information related to fermentation devices. The configuration of such devices ranges from single-vessel equipment (i.e., a single bioreactor) to more complex arrangements such as biolectors with multiple wells or arrays of multiple mini-bioreactors. MIFD was designed to support the recording of information related to most equipment configurations that are currently used within IB experiments and fermentations through integrating the concepts of device, vessel, and component in a hierarchical structure (Fig. 3). The device metadata subset refers to the top-level information describing fermentation equipment, while the vessel and component are associated with the fermentation vessel and its sensors or actuators, respectively. Some metadata terms included in MIFD are mapped to existing ontologies, while the remaining are designed *de novo* to accommodate the needs of IB projects. We believe that the proposed models will significantly enhance the process of integrating FAIR principles in the IB field, while facilitating easier adoption by the community by extending well-established standards and related ontologies.

## Design Considerations

### Ease of use

To facilitate ease of use of the proposed models as well as facilitate their future extension, 2 separate supplementary Excel files are provided containing all necessary information about MIFE (Supplementary File 1) and MIFD (Supplementary File 2). Each spreadsheet is split into distinct sheets providing information about the metadata terms at each hierarchical metadata level.

Both files have an identical structure, aiming to depict the models from an ontology-based perspective, ready to be parsed by our Python package that enables their programmatic use by creating a Linked Data Modeling Language (LinkML) schema [55]. The first sheet is named “metadata_levels” and provides a thorough description of each hierarchical level in MIFE (investigation, study, observation unit, sample, and assay) and MIFD (device, vessel, and component). Within the “metadata_levels” sheet, each row refers to a specific metadata level (denoted by the “metadata level” column), while the columns provide information regarding the purpose of each metadata level (“description” column); the object of the matching existing ontology concept (“object” column), if any; and what ontological class from an existing ontology it extends (“extends” column). The remaining sheets correspond to each hierarchical metadata level of MIFE and MIFD, where each row refers to a metadata term (denoted by the “term” column), and every column stores information about the relevant term such as which metadata level it belongs to (“metadata level” column); its data type (“value syntax” column); a description of its purpose with an example (“definition” and “example” columns); the required level of strictness (“strictness” column, e.g., “mandatory,” “optional”); how the term can be used to link concepts (“predicate” column); the object of a matching, existing ontology concept (“object” column), if any; and, whether it is a multivalued term or not (“multivalued” column). In the case of MIFE and the observation unit and sample sheets specifically, there exists an additional column, named “preferred unit,” which describes the preferred measurement units for certain terms.

To showcase the usefulness of the proposed minimum information approach, we have integrated MIFE within both FAIR Data Station (FAIR DS) [40] and an instance of FAIRDOM-SEEK [56–58], the IBISBA Knowledge Hub [59]. These tools facilitate metadata generation and validation (FAIR DS), as well as findability and accessibility, by providing a user-friendly way to structure and catalog metadata (FAIRDOM-SEEK). Users can directly visit the FAIR DS web portal [60], select the MIFE package, and test the MIFE integration by generating the relevant FAIR DS template and using it to validate their own metadata [40]. The validated metadata can be output as an RDF file that can then be imported into FAIRDOM-SEEK instances running version 1.17.0 or higher. Please note that a number of terms in MIFE are synonymous with terms used in these tools; where this occurs, the tool’s own term is prioritized over the term used in MIFE.

### Minimum information for fermentation experiments

To facilitate the needs of IB related projects, MIFE was developed by extending ISA to also include the concepts of observation units and samples (Fig. 2). The observation unit includes a set of metadata terms that specifically describe an experimental run using some fermentation equipment, while the sample concept refers to an entity (i.e., a small volume of the culture) taken from an observation unit at any stage of the fermentation process. In this extended ISA hierarchy, a single investigation may be associated with multiple studies, each study with multiple observation units, each observation unit with one or more samples, and each sample with multiple assays. MIFE metadata terms are described in Supplementary File 1. In total, 89 terms in MIFE were mapped to existing KOSs such as OM [61], PO2 [62], and JERM [39], among others [19, 34, 35, 41, 46, 63–65] (versions from May 2025).

### Minimum information for fermentation devices

To the best of our knowledge, up to the conclusion of the current study, the literature lacked a MIM to thoroughly describe the equipment configuration typically encountered in bioindustry and fermentation-based processes in general. There are published ontologies that model sensors [35, 41], but without considering the hierarchical configuration of IB equipment, such as biolectors with multiple wells or devices that can host many mini-bioreactors [66] or microbioreactors [67]. MIFD is the metadata schema that we propose for modeling fermentation devices, their components, and all the intricate hierarchical dependencies involved in the aforementioned equipment configurations (Fig. 3). The concept of device refers to the top layer of the cultivation equipment abstraction. Devices can host a single or have slots that can host multiple fermentation vessels. The concept of vessel reflects individual fermentation units that can be of bioreactor, microwell, or flask types. The component category describes sensors and actuators that are typically attached to fermentation vessels. In the proposed MIFD hierarchy, a device can host 1 or more vessels, and multiple components may be included into a single vessel. MIFD metadata terms are thoroughly described in Supplementary File 2. Similarly to MIFE, 26 terms in MIFD were mapped to existing ontologies (from versions available in May 2025) related to the IB, such as SOSA [34], SSN [35], and OM [61], among others [68, 69], to facilitate interoperability with community standards and effortless adoption by scientists in the field.

### Ontology and FAIR data station

The concept of MIMs is tightly connected to efforts in enabling reproducible science, an aspect of science that has attracted significant attention over the past 2 decades [70]. However, to be exploitable, structured metadata schemas such as MIMs must be incorporated into a usable format, such as user-friendly software that researchers can use to organize their experiments, generated data, computational analyses, and/or any other effort to share with the scientific community. Furthermore, alongside being human readable, MIMs need to be resolvable by software systems in a form that is computationally tractable; that is, they must be unambiguous and contained within machine-readable formats. By standardizing key experiment and equipment terms in both human and machine-readable formats, it will become far easier to not only reproduce or reuse existing datasets but also integrate data from multiple sources and automate data-processing pipelines for data analytics, facilitating advanced tasks like machine learning, digital twin simulations, and semantic querying over large datasets. In other words, consistent naming and structure are essential for any data-driven or AI-based workflow.

Therefore, to facilitate the programmatic use of the MIMs presented here, a LinkML schema encompassing the proposed terms for MIFE and MIFD was created. LinkML is not tied to a specific format or serialization, and thus it promotes interoperability across data formats and systems. Once defined, a LinkML schema can be transformed into multiple representations—including JSON Schema, OWL, SQL Database Schemas, and Python Data Classes. The LinkML representation was developed using a semi-automated process: an initial document was automatically generated, after parsing Supplementary Files 1 and 2, and was manually refined to remove extraneous artifacts. The resulting schema is available at the GitLab repository of the project. Because the ontology can be generated computationally, as new terms and relationships are required or the same terms and relationships evolve as needs change, the underlying ontology can be re-created with minimal manual input. The IB community is encouraged to submit requests for adding new or updating existing terms through the issue tracker of the GitLab repository.

In addition, researchers who wish to avail themselves of MIFE and MIFD can use FAIRDOM-SEEK [57] and FAIR DS, which have been adapted to allow metadata to be defined in line with the MIMs presented here. FAIR DS is a tool that can generate metadata templates in the form of Excel workbooks and, once filled in, validate the contents to ensure values entered by a researcher comply with the expected value. These templates enable researchers to systematically record experimental metadata in a FAIR and consistent manner, ensuring that metadata can be effectively shared, analyzed, and reused across experiments within and between facilities [40].

Key features of the FAIR DS include the following:

1. Automated Metadata Structuring: The system standardizes metadata capture, ensuring compliance with FAIR principles.
2. Interoperability with FAIRDOM-SEEK: The templates facilitate direct integration with FAIRDOM-SEEK, streamlining (meta)data sharing and collaboration.
3. Customizable Metadata Fields: Users can tailor the templates to accommodate domain-specific requirements while maintaining consistency across datasets.
4. Ontology Integration: The system leverages controlled vocabularies and ontologies to enhance data interoperability and semantic consistency.

To ensure data are findable, a key principle of FAIR, users can upload the validated metadata files from FAIR DS to FAIRDOM-SEEK. FAIRDOM-SEEK is an open-source web-based platform that adheres to the FAIR principles [2] for cataloging and sharing heterogeneous scientific research datasets, models or simulations, processes, and research outcomes. It was initially introduced as a tool that facilitates the sharing and integration of data in the systems biology domain, but it was quickly adopted by other domains. To showcase the seamless and automated integration of the work presented here within FAIRDOM-SEEK and similar research data management platforms, we integrated MIFE in FAIR DS [40]. Users can visit the FAIR DS web portal [60] and consult the relevant tutorials to generate a (meta)data workbook template using MIFE, fill in experimental metadata, and then subsequently validate them with FAIR DS [40] and proceed with uploading them to relevant data management platforms (e.g., IBISBA Knowledge Hub [71]). To demonstrate the usability of MIFE, Supplementary File 3 is a FAIR-DS–compliant MIFE template filled with metadata information. This file is based upon the dataset provided as part of the Industrial-scale Penicillin Simulation (IndPenSim) [72, 73], as it provides a suitable use-case that would be the equivalent of large, industrial-scale fermentations (e.g., [28]).

### Provenance

In addition to providing robust metadata schemas for IB bioprocesses, this work aimed to design MIFE and MIFD in a manner that enables alignment with and seamless integration into ongoing provenance standardization efforts. The Common Provenance Model (CPM) [9, 31] is a data model for provenance representation based on PROV-DM, which serves as an open conceptual foundation for the ISO 23494 Biotechnology—Provenance Information Model for Biological Material and Data series [9]. According to CPM, the most essential part of provenance is a *provenance backbone*, which is essentially a chain of standardized documentation of inputs (*backward connectors*) and outputs (*forward connectors*) of a documented process, also referred to as the *main activity*. Any other domain-specific information, including information about the subprocesses of the main activity, is appended to the backbone in a standardized way. As IB bioprocesses that are the main subject of the MIFE are typically only a single step in a broader workflow—starting from sample collection and processing, through a fermentation experiment, ending with the generated data processing and analysis—we see it important to be able to put the information from the experiment onto a standardized provenance chain. Table 1 summarizes the elements of the MIFE and MIFD that we consider basic building blocks to be integrated with the CPM (i.e., how the MIFE-related processes can correspond to the main activity in the CPM).

**Table 1:** Connecting MIFE and MIFD with the CPM. List of MIFE terms representing a process that can be represented as the main activity of the CPM and a list of its potential inputs and outputs that can be represented in the provenance backbone of the CPM. How specifically this mapping is implemented on the level of the CPM is dependent on the purpose of provenance collection (e.g., traceability, reusability, quality assessment, and reproducibility).

CPM main activity	CPM inputs (backward connectors)	CPM outputs (forward connectors)
Observation unit	Sample, vessel, protocol, feed materials, chemical compounds	Data for analysis, (technical) logfiles
Sample processing	Sample	Processed sample(s)
Sample collection	Source	Sample

## Conclusions

Since the early 21st century, the world has experienced an unprecedented rate of technological advancements, facilitating the constant emergence of breakthroughs across virtually every scientific discipline, in both academia and the private sector. Consequently, these technological advancements are fueling the Big Data era with an ever-increasing stream of heterogeneous data that comes in various, often not machine-readable, forms. Voluminous data can provide the ideal substrate for AI and XAI approaches to grow and accelerate even further knowledge discovery and technological innovation.

However, access to unlimited data cannot, on its own, guarantee the development of trustworthy models, since frequently, the information describing the data, also known as metadata, has been repeatedly proven to be equally valuable, if not more valuable than the actual data. MIMs and ontologies have been an integral part of data management and sharing, as well as in facilitating interoperable science. Meticulously designed MIMs not only promote FAIRification in science and efficient business operation in the private sector but also empower AI to achieve its full potential.

As a field that has been the hotspot of intense multidisciplinary research with significant industrial applications, IB could not deviate from the aforementioned norm. The technological innovations have transformed the field with equipment that can record data of fermentation experiments at an unprecedented scale, producing voluminous data in real time. Understanding bioprocesses and developing accurate modeling procedures that can facilitate informed decision-making in IB requires not only abundant and trustworthy data but also comprehensive data annotation in the form of rich metadata.

In this study, we propose a set of metadata schemas, MIFE and MIFD, specifically designed to promote FAIR principles in IB. Additionally, the structural dependencies between the subsets of terms of these MIMs and the integration with FAIR DS, as well as the implemented ontology, offer a novel approach for data standardization and annotation. Most terms were mapped to existing ontologies to facilitate interoperability with existing infrastructures and well-established standards while enabling easier adoption of the proposed MIMs by the community. The integration with FAIR DS, specifically, can significantly enhance user experience by automating (meta)data validation prior to converting it to machine-readable formats and using it for downstream modeling tasks. Additionally, FAIR DS–based validation ensures the incorporation of data and metadata into FAIR-inspired repositories, such as FAIRDOM-SEEK.

Even though the purpose of MIMs is to distill the necessary information for describing data to a minimal set of metadata, a different strategy was selected in this study. Both MIFE and MIFD include an extensive list of metadata terms that is divided into 3 main categories: (i) mandatory, (ii) recommended, and (iii) optional. The mandatory terms correspond to the minimum information required to describe fermentation data, while the recommended and optional terms allow for a more thorough type of management. Especially in the case of the observation unit subset of MIFE terms, an exhaustive list of terms is provided, most of which are optional. Filling a template with this many terms might seem daunting for day-to-day operations, hence the categorization into mandatory, recommended, and optional terms. Although MIFE and MIFD contain a comprehensive set of metadata terms and they exhibit a generic hierarchical structure design that can extend their application beyond IB-related fermentations, there may be cases where these models prove insufficient. In simple terms, a MIM cannot include a term for every possible concept. The development of new MIMs and the extension of existing ones should become a community effort that will lead to the development of a robust foundation for open science and (meta)data standardization.

Despite the aforementioned limitations, the field of IB and any fermentation domain in general can significantly benefit from efforts that attempt to offer (meta)data standardization, such as MIFE and MIFD, in a multifaceted way. Transitioning from basic research to industrial-scale applications can be accelerated, and AI can be seamlessly integrated into every step of this process. Interoperable data accompanied by rich metadata can accelerate knowledge discovery and enable the exploitation of explainable AI methods to understand the complex underlying mechanisms of bioprocesses.

## Availability of Source Code and Requirements

Project name: bioindustry-4.0/mim_ontology

Project homepage: https://gitlab.com/bioindustry-4.0/mim_ontology

Operating system: Windows/Linux/macOS

Programming language: Python (3.11)

Other requirements: FAIR Data Station and the following Python libraries: graphviz, linkml, linkml-runtime, numpy, openpyxl, pandas, PyYaml, rdflib, and ruamel.yaml

License: GPL-3.0 license

## Supplementary Material

giag038_Supplemental_Files

giag038_Authors_Response_To_Reviewer_Comments_original_submission

giag038_Authors_Response_To_Reviewer_Comments_revision_1

giag038_GIGA-D-25-00236_original_submission

giag038_GIGA-D-25-00236_Revision_1

giag038_GIGA-D-25-00236_Revision_2

giag038_Reviewer_1_Report_original_submission Reviewer 1 -- 7/15/2025

giag038_Reviewer_2_Report_original_submission Reviewer 2 -- 9/4/2025

giag038_Reviewer_2_Report_revision_1 Reviewer 2 -- 1/6/2026

## Data Availability

All resources described in this study are publicly available. The ontology documentation (MIFD and MIFE) is available here [74]. BioPortal serves as the ontology repository for MIFD [75] and MIFE [76]. The information deposited in the web servers is under the GPL-3.0 license. FAIR Data Station is accessible through its web portal [60] and also hosts validation tutorials [77]. MIFE is integrated with IBISBA Knowledge Hub [59]. As of the date of submission, Supplementary File 3 is compatible with FAIR-DS version 1.2.20-dev. For the most up-to-date version, visit the GitLab repository of the project [55]. The metadata models presented in this study are published under the GNU General Public License (GPL) version 3.0.

## Additional Files

**Supplementary File 1**: MIFE template

**Supplementary File 2**: MIFE template

**Supplementary File 3**: MIFE template

## Abbreviations

AI: artificial intelligence; BFO: Basic Formal Ontology; ChEBI: Chemical Entities of Biological Interest; CPM: Common Provenance Model; EBO: Environmental Biorefinery Ontology; EFO: Experimental Factor Ontology; ENVO: Environment Ontology; FAIR: Findable, Accessible, Interoperable, and Reproducible; FAIR DS: FAIR Data Station; FOAF: Friend-Of-A-Friend; IB: industrial biotechnology; ISA: investigation, study, assay; JERM: Just Enough Results Model; JSON: javascript object notation; KOS: knowledge organization systems; LinkML: Linked Data Modeling Language; MIAME: minimum information about a microarray experiment; MIFE: minimum information for fermentation experiments; MIFD: minimum information for fermentation devices; MIM: minimum information models; MIRIAM: minimum information requested in the annotation of biochemical models; MIxS: Minimum Information about any (x) Sequence; ML: machine learning; OBI: Ontology for Biomedical Investigations; OBO: Open Biological and Biomedical Ontology; OESO: Ontology of Experimental Scientific Objects; OM: ontology of units of measure; ORCID: Open Researcher and Contributor Identifier; OWL: web ontology language; PO2: Process and Observation Ontology; PROV-DM: PROV data model; PROV-O: PROV ontology; RDF: Resource Description Framework file format; SOSA: Sensor, Observation, Sample, and Actuator; SSN: Semantic Sensor Network; SQL: structured query language; XAI: explainable AI.
