# Supplementary material for: MIFE and MIFD: Minimum information for fermentation experiments and devices
Source: Gigascience. 2026 Apr 9;15:giag038. doi: 10.1093/gigascience/giag038 (PMC13184968; doi:10.1093/gigascience/giag038)
Supplement: giag038_GIGA-D-25-00236_Revision_1 [file giag038_giga-d-25-00236_revision_1.pdf]

GigaScience

MIFE and MIFD: Minimum information for fermentation experiments and devices

--Manuscript Draft--

**Manuscript Number:**	GIGA-D-25-00236R1
**Full Title:**	MIFE and MIFD: Minimum information for fermentation experiments and devices
**Article Type:**	Technical Note
**Funding Information:**	Horizon 2020 (101094287) — Not applicable<br>Dutch Research Council (NWO: 184.035.007) — Not applicable

Abstract:

Background

As the technological advancements of the early 21st century are pushing industrial biotechnology (IB) into the realm of Big Data driven innovation, the requirement for trustworthy data management, annotation and standardization is emerging as a necessity. Minimum information models (MIMs) have long been used across disciplines as the backbone of good data management practices by providing the scaffold upon which standardized recording of metadata can adequately and succinctly describe an under-study phenomenon.

Findings

Here we present a minimum set of metadata, named the minimum information for fermentation experiments (MIFE) and devices (MIFD), that has been specifically designed to accommodate the data management and annotation needs of IB related fermentation experiments. Although the proposed schema is tailored to IB applications, MIFE and MIFD builds upon well-established models and community standards to facilitate easier integration to existing infrastructure and easier adoption by the community, and aims to integrate Findable, Accessible, Interoperable and Reproducible (FAIR) principles in the IB field. In addition, the integration with FAIR Data Station (FAIR DS), a tool that offers metadata validation and enables the automated uptake of (meta)data from data management repositories such as FAIRDOM-SEEK, is showcased. The proposed models are accompanied by a Python package that enables their programmatic use by creating a Linked Data Modeling Language (LinkML) schema that can fuel subsequent analyses.

Conclusions

Through the promotion and simplification of knowledge discovery we believe that MIFE and MIFD can accelerate the application of state-of-the-art artificial intelligence (AI) methods and the adoption of explainable AI (XAI) to better understand bioprocesses at scale.

**Corresponding Author:**	Georgios K. Georgakilas, Ph.D.<br>Athena Research and Innovation Center in Information Communication and Knowledge Technologies: Athena Ereunetiko Kentro<br>Marousi, Attiki GREECE
**Corresponding Author Secondary Information:**	
**Corresponding Author's Institution:**	Athena Research and Innovation Center in Information Communication and Knowledge Technologies: Athena Ereunetiko Kentro
**Corresponding Author's Secondary Institution:**	
**First Author:**	Georgios K. Georgakilas, Ph.D.
**First Author Secondary Information:**	
**Order of Authors:**	Georgios K. Georgakilas, Ph.D.

Powered by Editorial Manager® and ProduXion Manager® from Aries Systems Corporation

	Brett Metcalfe
	Ariane Bize
	Matthew Crowther
	Emilie Fernandez
	Susana María Alonso Villela
	Stuart Owen
	Rudolf Wittner
	David Camilo Corrales
	Anselm von Gladiss
	Peter Blomberg
	Munazah Andrabi
	Cesar Arturo Aceves Lara
	Hans Mattila
	Marilyn Wiebe
	Theodore Dalamagas
	Jasper J. Koehorst
**Order of Authors Secondary Information:**	
**Response to Reviewers:**	We would like to thank both of the reviewers for their invaluable suggestions and feedback on the original version of the manuscript. We believe that by addressing the comments, there was a significant improvement in how our work is presented.<br>The reviewers’ comments can be found below in italics followed by a (detailed) response. Any adjustments on the manuscript have been colored in red.<br>Reviewer #1<br>The paper presents two complementary metadata schemas for describing fermentation experiments and the devices used in them. The topic is relevant and timely. As the authors argue, the definitions of commonly used metadata schemas align with the FAIR principles and contribute to community convergence. The importance of proper metadata, as defended by the authors, cannot be understated, which makes this paper particularly interesting.<br>However, I consider that this paper, in the current state, has two main issues: (1) the lack of test and validation and (2) contradiction on the purpose of providing a minimal information model.<br>Regarding 1, the authors presented motivation for such schemas, discussed related work and provided information about both MIFE and MIFD. However, there is no information about how the design considerations were defined and how the solution (the two metadata schemas) has been validated in real-world use cases and/or by the community. At least a test should have been described. As stated in the authors' submission instructions for GigaScience technical note papers (https://academic.oup.com/gigascience/pages/technical_note), "The tool or method needs to have been tested,...".<br>The information models presented in our study are part of the wider efforts of the BIOINDUSTRY4.0 consortium (https://www.bioindustry4.eu/) to advance the technological landscape of bioindustry in the EU. This consortium is composed of multiple institutes throughout Europe, bringing together highly interdisciplinary teams such as computer scientists, bioinformaticians, biotechnologists, biologists, and chemical engineers, among others.<br>MIFE and MIFD are the culmination of a two year effort of bringing this community together through workshops and webinars, reaching a consensus on the design requirements of structured metadata related to fermentation experiments. When writing the manuscript, we believed that the information about the aforementioned process would not provide any added value to the text, therefore we omitted it because it is irrelevant to the value and applicability of the models. However, if this is an important piece of information that should be in the manuscript, we could add it in a subsequent revision.

Powered by Editorial Manager® and ProduXion Manager® from Aries Systems Corporation

We agree with the reviewer that a usage example is currently missing from the manuscript. We added an additional supplementary file (Supplementary File 3) of a FAIR DS-ready template showcasing the usage of MIFE for sharing fermentation metadata. The file is ready to be validated by FAIR DS and then uploaded to FAIRDOM-SEEK (https://seek4science.org/) compliant platforms such as the IBISBA Knowledge Hub (https://hub.ibisba.eu/).

The second main issue is the term minimal information model, which is used in the title and throughout the paper. In my understanding, a minimal information model's purpose is to, as the term suggests, provide the minimal possible number of properties to describe a particular entity. And it seems that the authors agree with this understanding. In the conclusion section, the authors state that "Even though the purpose of MIMs is to distil the necessary information for describing data to a minimal set of metadata, a different strategy was selected in this study.". Then why are the metadata schemas called Minimal Information for Fermentation and Devices? Using a MIM to thoroughly describe something seems like a contradiction. Certainly, if we only consider the mandatory properties, one could argue that they constitute a minimal information model. But then I don't think that the names of the schemas are appropriate, as only a small subset of them are, in fact, MIMs.
We would like to thank the reviewer for the insightful comment. We agree that, conceptually, a Minimal Information Model (MIM) is meant to capture the essential set of metadata needed to describe a particular entity.
However, in biotechnology, the range of entities and processes that can be described is extremely broad. The discipline spans molecular and cellular studies, enzyme and metabolic engineering, systems and synthetic biology, as well as fermentation technology, bioprocess and chemical engineering, catalysis, and eco-design.
Because of this diversity, what constitutes “minimal information” is highly context-dependent: the core metadata required to describe a microbial strain, for instance, differ fundamentally from those required to describe a fermentation process, a bioreactor, or an analytical device.
Our intention was therefore not to use the term MIM in its narrowest sense, but to extend its logic to multiple biotechnological contexts — each with its own “minimal” set of descriptors needed for reproducibility, interoperability, and data reuse. The terms Minimal Information for Fermentation (MIF) and Minimal Information for Devices (MID) thus reflect this adaptation: they preserve the MIM philosophy while acknowledging the multi-scale and multi-disciplinary nature of biotechnology.
Additionally, the development of MIFE and MIFD was initiated based on the need of standardizing fermentation metadata for data interoperability within the BIOINDUSTRY4.0 consortium, and due to the lack of IB-specific metadata standards in the literature. In early stages of the development both models included the metadata terms marked as mandatory, since the initial objective was to prepare a minimum set of terms for describing IB data. However, as the development was progressing and the BIOINDUSTRY4.0 consortium was exchanging opinions on data interoperability and modelling requirements through workshops and webinars, it became apparent that a richer and more flexible schema was required. To remain faithful to our initial objective of creating minimum information models for IB, we decided to divide terms into mandatory, recommended and optional.

Other minor comments include:
- In the "Minimum information models" section, the authors MIMs allow a community to adopt specific criteria deemed essential, required, or mandatory...". This is true of any data model, minimal or not. Almost every data modeling approach, e.g., UML class diagram, ER, etc., has a way of specifying cardinality where a minimal cardinally of 1 means mandatory and of 0 means optional.
The mandatory terms justify the reasoning behind the word minimum, while the remaining terms add the flexibility required by scientists involved in day-to-day research within the IB field. For instance, description of the purpose of the fermentation run is an essential descriptor for any fermentation process, but parameters such as what substance is the strain resistant to, or equipment used to feed the system may be context-dependent and therefore classified as recommended or optional.

- In section "Minimum information models in industrial biotechnology", the authors state "To make sense of IB (experimental) data and ensure that users of such data understand what will occur, is occurring, and has occurred (Figure 1) necessitates that

	a data consumer is provided with:...". Is this necessity based on community consensus, expert opinion or some other requirement? And is it true for any application of IB data?<br>As mentioned in the previous comments, this study is an effort of the BIOINDUSTRY4.0 consortium to establish a set of metadata standards for fermentation data. Through workshops and webinars, scientists from different disciplines reached a consensus of how bioprocess metadata should be organized for streamlining downstream analyses, such as establishing kinetic, or genome-scale models, machine learning model training and soft-sensor applications for digital twins. The goal of IB data analytics is to provide a better understanding of the utilized microbial factories, leading to improved performance of bioprocesses and optimized product yield. This process is frequently hindered by the lack of metadata standards, presenting a significant obstacle to chemical engineers and computer scientists that attempt to develop predictive models and digital twin infrastructures.<br><br>- The title of the section "Overview of existing minimum information models" seems inadequate as it describes ontologies and vocabularies that are, arqueable, not minimal, e.g., BFO, SOSA, SSN, etc.<br>In this section we attempted to provide an overview of knowledge organization systems that are potentially relevant to IB but not specific to IB, at least to the extent that is required by the IB community. We agree with the reviewer that since these models are not minimum mode, the title of the section should be adjusted accordingly. Thus, we changed the title to “Overview of existing knowledge organization systems”.<br><br>- In the sentence starting with "The application core Ontology of Experimental Scientific Objects (OESO-CORE) reuses...", what are the definitions of core and application ontologies? It doesn't seem that FOAF, SOSA and PROV-O are at the same level, and one could argue that, at least, FOAF and PROV-O are not upper-level ontologies. Maybe core ontologies.<br>We agree that SOSA etc. are not on the same level as BFO (upper-level), and are rather core ontologies. We have corrected the text in the article as follows: “It reuses concepts from upper-level and core ontologies (e.g., BFO, SOSA)” and “The application core Ontology of Experimental Scientific Objects (OESO-CORE) reuses concepts from core ontologies (e.g., FOAF, SOSA, PROV-O)”.<br><br>- Subsection "Ease-of-use": from the Gitlab files, it seems that the url column represents the object type or the datatype.<br>We would like to thank the reviewer for the apt remark. It was intended that the url column would be used for generating the linkML file. However, the name "url" does not indeed cover the heterogeneity of this column. To remain general, we renamed it as "object" (it refers to the triplet structure, where we have a subject, here corresponding to the column term, a predicate (column predicate), and an object (the url column that was renamed to object). The object column basically represents the value of the property: a resource or a literal (i.e., a string, number, date)<br><br>Reviewer #2<br>The manuscript describes the development of two new standards, minimum information for fermentation experiments (MIFE) and devices (MIFD), addressing challenges in these communities to report data within a standardized framework.<br>Well written, sufficient justification provided in the background section.<br>-- when providing examples, remove 'etc<br>We removed ‘etc from the relevant parts of the text.<br><br>-- Overview of existing minimum information models<br>In this section, I would have expected consideration of OBI: Ontology for Biomedical Investigations. Are there portions of OBI that would be pertinent to the developed standards?<br>We would like to thank the reviewer for the apt remark. Indeed, even though OBI is developed according to Biomedical standards, some individual concepts could be applicable to IB as well. At an earlier stage of MIFE and MIFD development, we considered using OBI terms related to, i.e., studies and investigations. However, we decided to include relevant terms from other metadata schemas, whose domain seemed closer to industrial biotechnology than the biomedical field. We have added relevant text mentioning OBI in the “Overview of existing knowledge organization systems” section.

-- Briefly outline methods utilized to identify/map individual terms within the standards to other standards, and how these mappings/re-use of terms were integrated.
To map the terms, we conducted research in various ontology portals, such as Bioportal and Agroportal. We considered broader and narrower concepts, as well as definitions. We used SKOS relationships (e.g., exact match) to integrate these mappings into the MIMs. These relationships are visible in the MIM publication on Bioportal, within each class.

It is noted in the Aims and Objective section:
Some metadata terms included in MIFD are mapped to existing ontologies.
-- How many terms, from which ontologies? What version of each ontology were the terms mapped to?
The ontology versions used were those from May 2025. Regarding the ontologies used for the mapping, they are mentioned in the manuscript in the sections "Minimum information for fermentation experiments" and "Minimum information for fermentation devices" (and references cited therein). In total, 89 terms in the MIFE and 26 in MIFD were mapped to other KOS concepts. We added relevant information in the two sections mentioned above.

--In the Design Considerations section, it is noted:
-- "what ontological class from an existing ontology it extends" and "the URL of a matching, existing, ontology concept ("url" column" --> Is the ID of the original term retained?
The classes of existing ontologies were mapped and not imported into the MIM, so the IDs of the original terms were not retained. MIFD and MIFE have their own ID system.

- Two edits for this sentence:
From: Minimum Information about any (X) Sequence (MIXS) and its subcategories from the Genomics Standards Consortium
to: Minimum Information about any (X) Sequence (MIxS) and its subcategories from the Genomic Standards Consortium
We made the proposed change into the relevant part of the text.

- Include the specific license associated with the standards.e.g., CCBY or CCO
-- Add this to the Availability statement.
We added a sentence specifying the licence associated to MIFE and MIFD in the "Availability of Source Code and Requirements" section.

-- when using e.g. -- always add a comma at the end, e.g.,
-- At the end of the Ontology and FAIR Data Station, there is a e.g. without the comma
We made the appropriate adjustments in the text.

-- Figure 4 - the text in the figure is blurry and the font size could be increased for readability
We reworked the font size in figure 4 to improve readability. We also provide all figures as separate files.

-- Conclusion
-- Is there a mention in the manuscript for gathering and integrating updates to the standards? How is this planned for ? Do you want users to submit issues to: https://gitlab.com/bioindustry-4.0/mim_ontology. Are there datasets in preparation for this standard? Where will the datasets be submitted ? How often will updates to the schema be made available, that is, what is the planned update cycle?

We have included an additional supplementary file (Supplementary File 3) that includes a FAIR DS-ready template, filled with metadata from a published dataset. This file is ready to be validated using FAIR DS and then uploaded into FAIRDOM-SEEK-compliant platforms such as the IBISBA Knowledge Hub. The IBISBAKhub has implemented the MIFE under extended metadata so any user of IBISBA can already submit using this standard. We added relevant text in the "Ontology and FAIR Data Station" section.
Since the schema is an integral component of the IBISBA RI metadata standards, updates will happen based on the needs of IBISBA. Regardless, the community can

	submit requests about additions or adjustments to the schema through the GitLab issue tracking system. The consortium will be assessing such requests and evaluating their integration in future releases, if future releases are needed.
**Additional Information:**	
**Question**	**Response**
Are you submitting this manuscript to a special series or article collection?	No
**Experimental design and statistics**<br><br>Full details of the experimental design and statistical methods used should be given in the Methods section, as detailed in our Minimum Standards Reporting Checklist. Information essential to interpreting the data presented should be made available in the figure legends.<br><br>Have you included all the information requested in your manuscript?	Yes
**Resources**<br><br>A description of all resources used, including antibodies, cell lines, animals and software tools, with enough information to allow them to be uniquely identified, should be included in the Methods section. Authors are strongly encouraged to cite Research Resource Identifiers (RRIDs) for antibodies, model organisms and tools, where possible.<br><br>Have you included the information requested as detailed in our Minimum Standards Reporting Checklist?	Yes
**Availability of data and materials**<br><br>All datasets and code on which the conclusions of the paper rely must be either included in your submission or deposited in publicly available repositories (where available and ethically appropriate), referencing such data using a unique identifier in the references and in	Yes

the “Availability of Data and Materials” section of your manuscript.<br><br>Have you have met the above requirement as detailed in our Minimum Standards Reporting Checklist?	
GigaScience has policies and guidelines in place for the use of generative AI-writing tools such as ChatGPT. If you have used such writing tools to assist with writing the manuscript this must be declared and cited in the text. Authors should not list AI-writing tools and other AI-assisted technologies as an author or co-author and should acknowledge that they are fully responsible for text generated or refined by AI-writing tools.<p><br><br>A summary of use (particularly in the introduction or among methods) needs to be included at the end of the paper, and the outputs should also be included as a supplementary file hosted in GigaDB or other open repositories. Please read our guidelines for more information. <p><br><br>By submitting to GigaScience, you are aware of the journal's AI-writing tools policy, and if you have declared use of such tools below, you have acknowledged this where appropriate in your manuscript and have made a summary of use and outputs available. <p><br><b>AI-assisted writing tools have been used in the preparation of this manuscript?	No

Revised Manuscript
Click here to access/download;Manuscript;revised manuscript.docx

MIFE and MIFD: Minimum information for fermentation experiments and devices

Georgios K. Georgakilas[1,*,#], Brett Metcalfe[2,3,4,*,□], Ariane Bize[5], Matthew Crowther[6], Emilie Fernandez[7], Susana Maria Alonso Villela[8], Stuart Owen[9], Rudolf Wittner[10], David Camilo Corrales[8], Anselm von Gladiss[11], Peter Blomberg[12], Munazah Andrabi[9], Cesar Arturo Aceves Lara[8], Hans Mattila[12], Marily Wiebe[12], Theodore Dalamagas[1] & Jasper J. Koehorst[2,13,□]

1. Information Management Systems Institute, ATHENA Research Center, 15125 Marousi, Greece.

2. Laboratory of Systems and Synthetic Biology, Department of Agrotechnology and Food Sciences, Wageningen University & Research, 6708 PB Wageningen, The Netherlands.

3. Department of Earth Sciences, Faculty of Science, Vrije Universiteit Amsterdam, 1081 HV Amsterdam, The Netherlands.

4. Amsterdam University College, 1098 XG Amsterdam, The Netherlands

5. Université Paris-Saclay, INRAE, PROSE, 92160 Antony, France.

6. Newcastle University, NE1 7RU Newcastle Upon Tyne, United Kingdom.

7. INRAE, University of Montpellier, LBE, 11100 Narbonne, France.

8. TBI, Université de Toulouse, CNRS, INRAE, INSA, 31077 Toulouse, France.

9. The University of Manchester, M13 9PL Manchester, United Kingdom.

10. BBMRI-ERIC, Neue Stiftingtalstrasse 2/B/6, 8010 Graz, Austria.

11. Institute for Computer Science, University of Koblenz, 56070 Koblenz, Germany.

12. VTT Technical Research Centre of Finland, PO Box 1000, 02044 VTT, Finland.

13. UNLOCK, Wageningen University & Research and Delft University of Technology, The Netherlands.

** Equal contribution.*

# To whom correspondence should be addressed.

□ To whom correspondence could also be addressed.

Abstract

Background

As the technological advancements of the early 21st century are pushing industrial biotechnology (IB) into the realm of Big Data driven innovation, the requirement for trustworthy data management, annotation and standardization is emerging as a necessity. Minimum information models (MIMs) have long been used across disciplines as the backbone of good data management practices by providing the scaffold upon which standardized recording of metadata can adequately and succinctly describe an under-study phenomenon.

Findings

Here we present a minimum set of metadata, named the minimum information for fermentation experiments (MIFE) and devices (MIFD), that has been specifically designed to accommodate the data management and annotation needs of IB related fermentation experiments. Although the proposed schema is tailored to IB applications, MIFE and MIFD builds upon well-established models and community standards to facilitate easier integration to existing infrastructure and easier adoption by the community, and aims to integrate Findable, Accessible, Interoperable and Reproducible (FAIR) principles in the IB field. In addition, the integration with FAIR Data Station (FAIR DS), a tool that offers metadata validation and enables the automated uptake of (meta)data from data management repositories such as FAIRDOM-SEEK, is showcased. The proposed models are accompanied by a Python package that enables their programmatic use by creating a Linked Data Modeling Language (LinkML) schema that can fuel subsequent analyses.

Conclusions

Through the promotion and simplification of knowledge discovery we believe that MIFE and MIFD can accelerate the application of state-of-the-art artificial intelligence (AI) methods and the adoption of explainable AI (XAI) to better understand bioprocesses at scale.

Background

The expectation of data sharing between, and within, institutions has begun to become the scientific norm in many disciplines of science[1]. With such endeavours being further promoted by initiatives led by individuals; institutions; funding and governmental agencies seeking to facilitate best practices through the adoption and promotion of the principles of both Findable, Accessible, Interoperable, Reusable (FAIR)[2] and open science initiatives[3]. That is because good data management practice is not just a bureaucratic (or academic) exercise but is crucial for cultivating new knowledge, facilitating data sharing, and ensuring reproducibility[4,5]. Such practices are even more essential for disciplines with tighter relations to the commercial domain where regulatory agencies (*e.g.*, for food, healthcare, drug, vaccine, and pharmaceutical development)[6,7] require that the provenance[8,9] of the product is sufficiently well known. Industrial biotechnology (IB), bioprocess engineering[10], and synthetic biology are examples of one such group of disciplines. IB endeavours to incorporate biological know-how with engineering principles, to utilise (un)modified organisms grown under relatively restrictive conditions within a vessel to either synthesise bio-based products and consumables or the building blocks towards them at industrial scale[10].

Minimum information models

As the adoption of good data management practices gathers pace, questions have now begun to switch from 'why' to 'what metadata' - (*i.e.*, data about data) - should be required alongside datasets (*e.g.*, [11–16]). One approach to ensure the recording of sufficient and relevant information is through the use of community defined minimum information models (MIMs)[17,18] that provide a minimum set of standardised metadata which are required to adequately describe an object or phenomenon under study. MIMs allow a community to adopt specific criteria deemed essential, required, or mandatory whilst giving a researcher the flexibility to describe, in detail, additional optional or recommended metadata if they so require. Here, mandatory, recommended, and optional metadata are defined as:

1. **Mandatory**. Aspects of metadata that the experimentalist is required to record and are crucial for understanding the data. For example, providing information about the microbial strain that was used in an experiment, the bioreactor conditions throughout a fermentation experiment with the information of each sensor.

2. **Recommended**. Aspects of metadata that are encouraged to be cataloged but are not mandatory and can be treated flexibly. For example, providing an Open Researcher and Contributor Identifier (ORCID) for an author is recommended to differentiate two or more John Smiths' but it is not strictly necessary as the same information can be gleaned elsewhere (*e.g.*, institution and email).

3. **Optional**. Aspects of metadata that may be selected, thereby providing flexibility to add additional information that the user deems pertinent but which is not required to understand the dataset. For example, additional information may be provided about an organism under cultivation such as the initial isolation, potential synonyms, and extended taxonomic information.

The benefit of a MIM is it allows focussing on the core information that a system's behaviour is based on. When analysing systems, reducing the amount of information with a MIM means reducing the number of parameters to analyse. In such a model - through reducing the complexity - fundamental

principles and key variables of a system can be identified better. Thus, MIMs help in analysing systems and testing hypotheses. In addition, MIMs not only facilitate data sharing and system analysis, but are a big step towards standardised and consistent documentation, and further motivate standardised experimental settings. Examples of such standardisation initiatives include the *minimum information about a microarray experiment* (MIAME)[19] and the *Minimum information requested in the annotation of biochemical models* (MIRIAM)[20]. This claim holds especially for IB, as MIMs ensure consistent metadata and thus, comparability of experimental results. In complex settings such as bioprocesses, clear guidelines on metadata collection reduces the number of missteps taken in development. Together with an improvement in experimental consistency this may help in replicating and scaling up processes, translating research and development into commercial production, and facilitating regulatory approvals.

Minimum information models in industrial biotechnology

To make sense of IB (experimental) data and ensure that users of such data understand what will occur, is occurring, and has occurred (**Figure 1**) necessitates that a data consumer should at least be provided with: i) information on the organism, any modifications, or particular strains, ii) information on the cultivation equipment used including type of sensors and actuators, iii) a description and information on the experimental set-up and protocol used, including set-points, iv) information and a description of (any) sampling, as well as the v) location and description of the data and data analysis. Furthermore, advancements in IB are predicted to allow for enhanced monitoring of bioprocesses through off-line, at-line, on-line, and in-line methods[21] and/or modeling of bioreactors via artificial intelligence (AI), machine learning (ML), and explainable AI (XAI) methods[22,23]. Such advancements should enable more intuitive steering of the bioprocess - so-called Digital Twins[24] - however, to achieve this requires that data and metadata become standardized and machine readable.

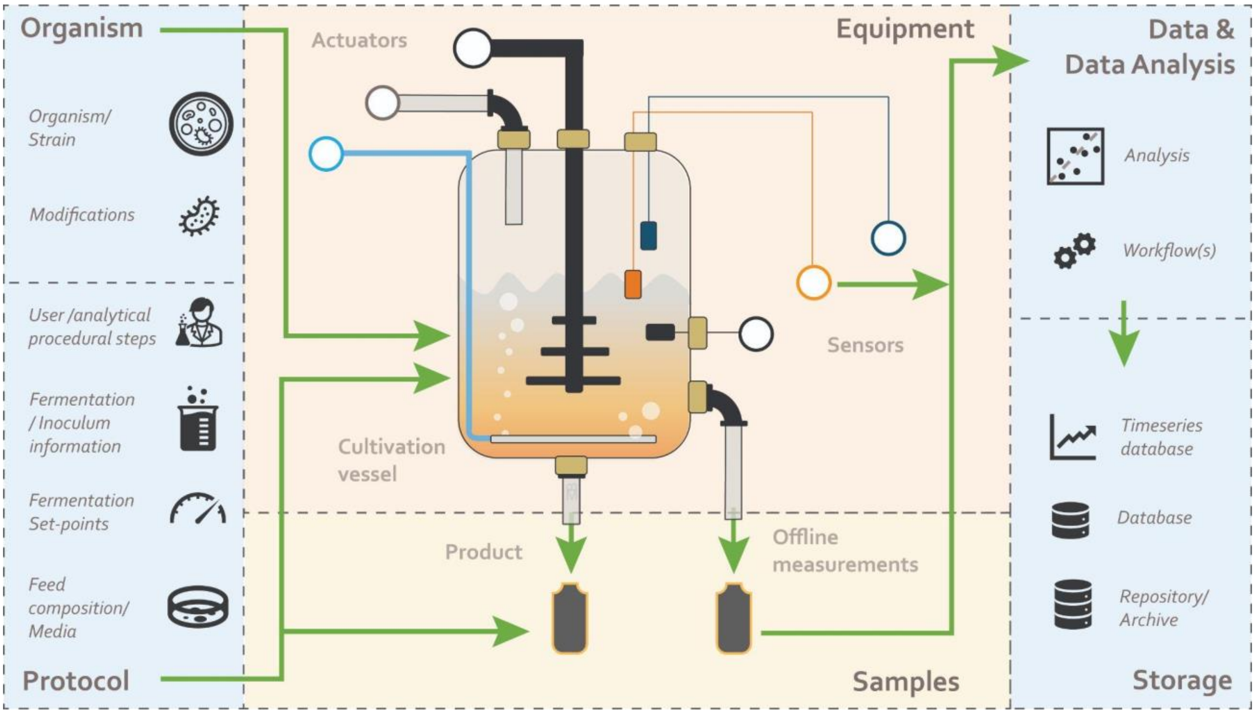

Figure 1. Information necessary to understand the data associated with an industrial biotechnology fermentation experiment. *Green arrows show the relationships between the various aspects of information.*

A secondary consideration is to ensure traceability which is handled by provenance, essentially information about entities, activities, and people involved in producing a piece of data or object[25]. As data and its precursors (*e.g.*, biological or environmental samples) are typically shared between organizations, each of the organizations can provide information only for a part of the described object's lifecycle. In turn, this results in a situation where complete documentation of the object's provenance is easily fragmented, as it is generated and can be managed by various heterogeneous institutions[26]. Such a scenario is becoming even more relevant in the context of the European strategy for data, namely the Common European Data Spaces[27] or AI Act[28]. To enable full traceability of the dataset history, it is essential to enable linking the various, fragmented, provenance pieces. Overcoming the challenges of fragmented provenance has been a focal point of provenance research in recent years[26], and is currently a subject of a standardization within the *ISO TC 276 Biotechnology WG 5 Data Processing and Integration*, under the *ISO 23494 Provenance Information Model for Biological Material and Data series*[9]. This series provides a domain-agnostic integrative horizontal framework for provenance.

Overview of existing knowledge organization systems

In contrast, MIMs are a crucial complement to such a provenance framework, as they add fundamental *domain-specific* information. Several existing MIMs and other types of knowledge organization systems (KOS) are relevant to the field of IB and the fermentations it encompasses. However, to our knowledge, none of the currently available ones comprehensively cover the general and specific concepts needed to describe IB experiments. For example, the Sensor, Observation, Sample, and Actuator (SOSA)[29] as well as Semantic Sensor Network Ontology(SSN)[30] ontologies are dedicated to sensors while PROV-O (the PROV ontology)[25] addresses provenance, and Friend-Of-A-Friend (FOAF)[31] focuses on describing persons and their relations. The Basic Formal Ontology (BFO)[32] developed by the Open Biological and Biomedical Ontology (OBO) Foundry is an upper level ontology, helping to ensure consistency, interoperability, and logical coherence across diverse ontologies and knowledge domains.

The Investigation, Study, Assay (ISA) abstract model[33] and associated isa-tools is widely used in the field of biology. On this basis, an ontology has also been created, named Just Enough Results Model (JERM)[34]. Whilst ISA represents a metadata framework for describing projects and associated experiments it does not provide an adequate model for describing the equipment used in those projects, equipment that is central to IB fermentations. The ISA framework also lacks a conceptual hierarchical level that describes observation units[35] (*e.g.*, a fermentation run), samples, fermentation devices and sensors among other items depicted in **Figure 1**. One alternative is the Experimental Factor Ontology (EFO)[36] that provides a systematic description of many experimental variables related to molecular biology but it also lacks many concepts specifically related to IB fermentation. For instance, it includes the concept of instrument, with 84 distinct specific instrument classes (*e.g.*, centrifuge, spectrophotometer); but other key instruments for industrial biotechnology experiments are lacking, such as mass flow controllers and bioreactor vessels.

Regarding the description of processes, the Process and Observation Ontology (PO2)[37] and its associated management tool are especially relevant. It reuses concepts from upper-level and core ontologies (*e.g.*, BFO, SOSA) and proposes generic concepts that can be specialized for domain ontologies. The concepts that can be specialized are component, step, attribute, process, material, method, and scale. PO2 however lacks domain-specific terms for IB fermentation processes. The application core Ontology of Experimental Scientific Objects (OESO-CORE)[38] reuses concepts from

core ontologies (*e.g.*, FOAF, SOSA, PROV-O) and enables the description of concepts such as organization and experimental context, types of environment, equipment and physical objects. Since it is not IB-oriented, it cannot fully support engineering and physical parameters critical to fermentation. The Environmental Biorefinery Ontology (EBO)[39] extends the core ontology PO2 and the OESO-CORE[38] to provide a framework for describing various aspects of environmental biorefinery processes. This includes the characterization of instruments, components, and processes involved in the transformation of biomass into valuable products. However, it has shortcomings in the description of strains, their induction and comprehensively describing the conditions of a fermentation experiment.

Another alternative is the Environment Ontology (ENVO)[40] that is used to represent knowledge about environments, environmental processes, ecosystems, habitats, and related entities. However, being mostly related to the environment, most ENVO terms that could be relevant for IB, such as bioreactor, actually refer to environmental biotechnology processes (waste or wastewater treatment) rather than IB. Alongside the IB-adjacent KOS presented above, a significant number of additional KOS are relevant to IB experiments, but restricted to specific aspects of IB. Non exhaustive examples are Chemical Entities of Biological Interest (ChEBI)[41], Minimum Information about any (X) Sequence (MIxS) and its subcategories from the Genomics Standards Consortium[42], MIMs for other types of experimental data[19,43,44] and the BacDive repository[45] that is relevant to reference strains, but does not yet include eukaryotes (*e.g.*, yeast). Finally, the Ontology for Biomedical Investigations (OBI)[46] is a rich ontology which describes scientific and/or clinical investigations; the protocols they utilise as well as instrumentation and materials; the generated data; and, the types of analysis performed upon the data. Although many individual concepts of OBI are relevant to the field of IB, its application to describe IB processes is limited because of its general orientation toward the biomedical field.

Aims and objectives

Therefore, as there is no MIM specifically relevant for the needs of IB, here we introduce the Minimum Information for Fermentation Experiments (MIFE) and Devices (MIFD) models that provide a set of standardized metadata schemas tailored to the field of IB (**Figures 2 and 3**). These proposed models - available as ontologies deposited in BioPortal[47] - were designed to thoroughly describe IB experiments by adopting terms from IB-adjacent MIMs and ontologies found in the literature supplemented with new terms that describe integral components of IB bioprocesses that are currently missing from existing models. Briefly, MIFE was developed by extending ISA[33] to also include the concepts of observation units[35,48] and samples that correspond to fermentation runs and samples taken from a fermentation vessel during or after a run is completed respectively. The hierarchical nature of the extended ISA structure behind MIFE is capable of supporting the management of entire projects by enabling the thorough recording of key information related to fermentation experiments (**Figure 4**). In addition to metadata regarding the purpose of the investigation or each experiment specifically and the people involved, MIFE also covers the operating conditions of a fermentation vessel, the strain used as a microbial cell factory, the samples taken throughout the process and any assay applied on these samples (**Figure 2**).

MIFE is complemented by MIFD, which is a hierarchical model for specifically capturing key information related to fermentation devices. The configuration of such devices ranges from single-vessel equipment (*i.e.*, a single bioreactor) to more complex arrangements such as biolectors with multiple wells or arrays of multiple mini bioreactors. MIFD was designed to support the recording

of information related to the vast majority of equipment configurations that are currently utilised within IB experiments and fermentations through integrating the concepts of device, vessel, and component in a hierarchical structure (**Figure 3**). The device metadata subset refers to the top level information describing fermentation equipment, while the vessel and component are associated to the fermentation vessel and its sensors or actuators respectively. Some metadata terms included in MIFD are mapped to existing ontologies, while the remaining are designed *de novo* to accommodate the needs of IB projects. We believe that the proposed models will significantly enhance the process of integrating FAIR principles in the IB field, while facilitating easier adoption by the community by extending well established standards and related ontologies.

Design Considerations

Ease-of-use

To facilitate ease-of-use of the proposed models as well as facilitate their future extension, two separate supplementary excel files are provided containing all necessary information about MIFE (**Supplementary File 1**) and MIFD (**Supplementary File 2**). Each spreadsheet is split into distinct sheets providing information about the metadata terms at each hierarchical metadata level.

Both files have an identical structure aiming to depict the models from an ontology-based perspective, ready to be parsed by our Python package that enables their programmatic use by creating a Linked Data Modeling Language (LinkML) schema, available at https://gitlab.com/bioindustry-4.0/mim_ontology. The first sheet is named "metadata_levels" and provides a thorough description of each hierarchical level in MIFE (investigation, study, observationUnit, sample and assay) and MIFD (device, vessel and component). Within the "metadata_levels" sheet, each row refers to a specific metadata level (denoted by the "metadata level" column) while the columns provide information regarding the purpose of each metadata level ("description" column), the object of the matching existing ontology concept ("object" column), if any, and what ontological class from an existing ontology it extends ("extends" column). The remaining sheets correspond to each hierarchical metadata level of MIFE and MIFD, where each row refers to a metadata term (denoted by the "term" column) and every column stores information about the relevant term such as which metadata level it belongs to ("metadata level" column); its data type ("value syntax" column); a description of its purpose with an example ("definition" and "example" columns); the required level of strictness ("strictness" column, *e.g.*, 'mandatory', 'optional'); how the term can be used to link concepts ("predicate" column); the object of a matching, existing, ontology concept ("object" column), if any; and, whether it is a multi-valued term or not ("multivalued" column). In the case of MIFE and the observationUnit and sample sheets specifically, there exists an additional column, named "preferred unit", which describes the preferred measurement units for certain terms.

To showcase the usefulness of the proposed minimum information approach, we have integrated MIFE within both FAIR Data Station (FAIR DS)[35] and an instance of FAIRDOM-SEEK[49–51], the IBISBA Knowledge Hub (https://ibisbahub.eu/). These tools facilitate metadata generation and validation (FAIR DS), and findability and accessibility by providing a user-friendly way to structure and catalogue metadata (FAIRDOM-SEEK). Users can directly visit the FAIR DS web portal (https://fairds.fairbydesign.nl/) select the MIFE package and test the MIFE integration by generating the relevant FAIR DS template and using it to validate their own metadata[35]. The

validated metadata can be output as an RDF file that can be then imported into FAIRDOM-SEEK instances running version 1.17.0 or higher.

Minimum information for fermentation experiments

To facilitate the needs of IB related projects, MIFE was developed by extending ISA to also include the concepts of observation units and samples (**Figure 2**). The observation unit includes a set of metadata terms that specifically describe an experimental run using some fermentation equipment, while the sample concept refers to an entity (*i.e.*, a small volume of the culture) taken from an observation unit at any stage of the fermentation process. In this extended ISA hierarchy, a single investigation may be associated with multiple studies, each study with multiple observation units, each observation unit with one or more samples and each sample can be connected with multiple assays. MIFE metadata terms are described in **Supplementary File 1**. In total, 89 terms in MIFE were mapped to existing KOS such as OM[52], PO2[53] and JERM[34], among others[18,29,30,36,41,54–56] (versions from May 2025).

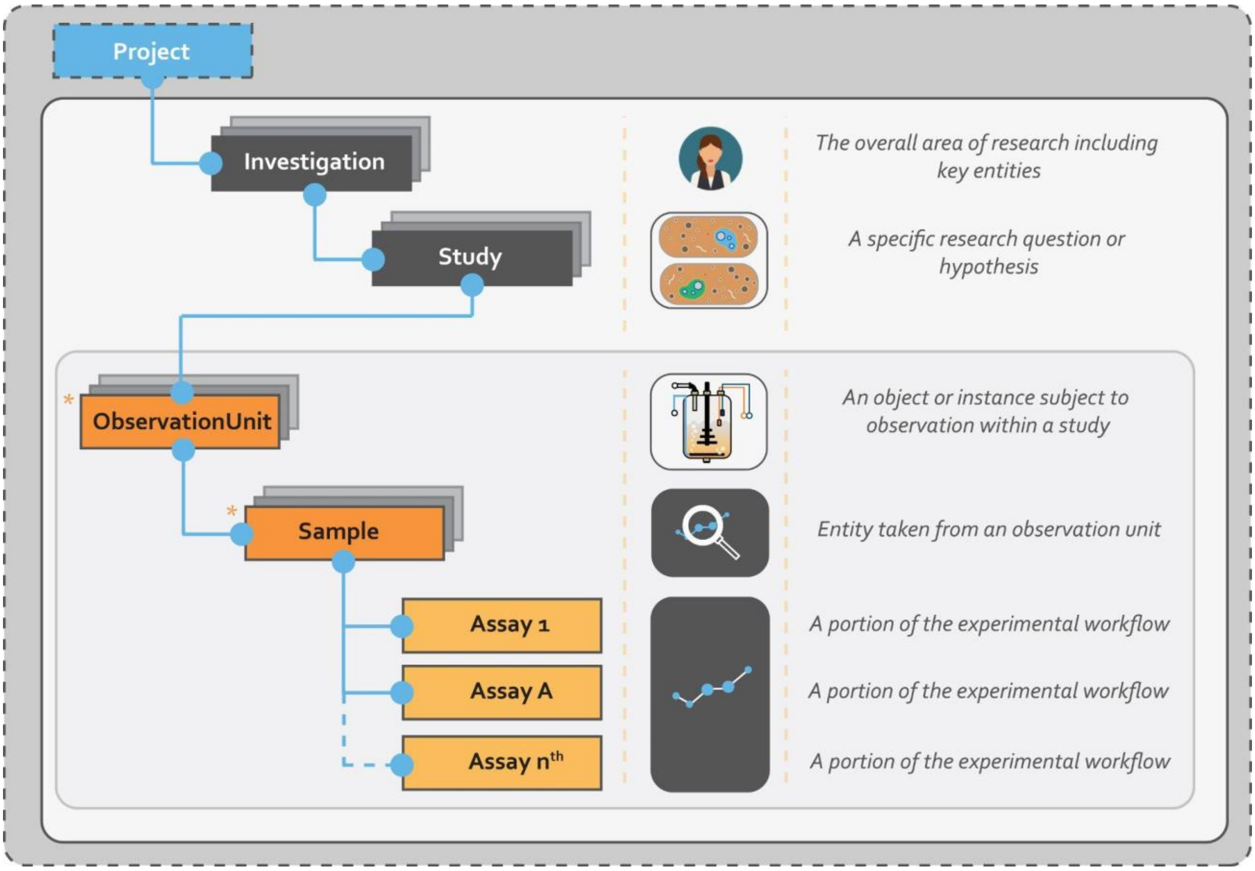

Figure 2. MIFE metadata hierarchy. *MIFE extends the ISA structure[33] by incorporating novel metadata terms related to the concepts of observation units and samples. The observation unit subset describes fermentation runs while sample refers to entities taken from the culture at any step of the process. Each investigation may include many studies, a study can be supported by multiple observation units from which culture volume entities can be taken to be processed with experimental assays. Levels with a * represent the extension[35] to ISA[33].*

Minimum information for fermentation devices

To the best of our knowledge, up to the conclusion of the current study, the literature lacked a MIM to thoroughly describe the equipment configuration that is typically encountered in bioindustry and fermentation-based processes in general. There are published ontologies that model sensors[30,36], however, without considering the hierarchical configuration of IB equipment such as biolectors with multiple wells or devices that can host many mini bioreactors[57] or microbioreactors[58]. MIFD is the metadata schema that we propose for modeling fermentation devices, their components and all the intricate hierarchical dependencies involved in the aforementioned equipment configurations (**Figure 3**). The concept of device refers to the top layer of the cultivation equipment abstraction. Devices can host single or have slots that can host multiple fermentation vessels. The concept of vessel reflects individual fermentation units that can be of bioreactor, microwell or flask types. The component category describes sensors and actuators that are typically attached to fermentation vessels. In the proposed MIFD hierarchy, a device can host one or more vessels, and multiple components may be included into a single vessel. MIFD metadata terms are thoroughly described in **Supplementary File 2**. Similarly to MIFE, 26 terms in MIFD were mapped to existing ontologies (from versions available in May 2025) related to the IB such as SOSA[29] and SSN[30] and OM[52], among others[59,60], to facilitate interoperability with community standards and effortless adoption from scientists in the field.

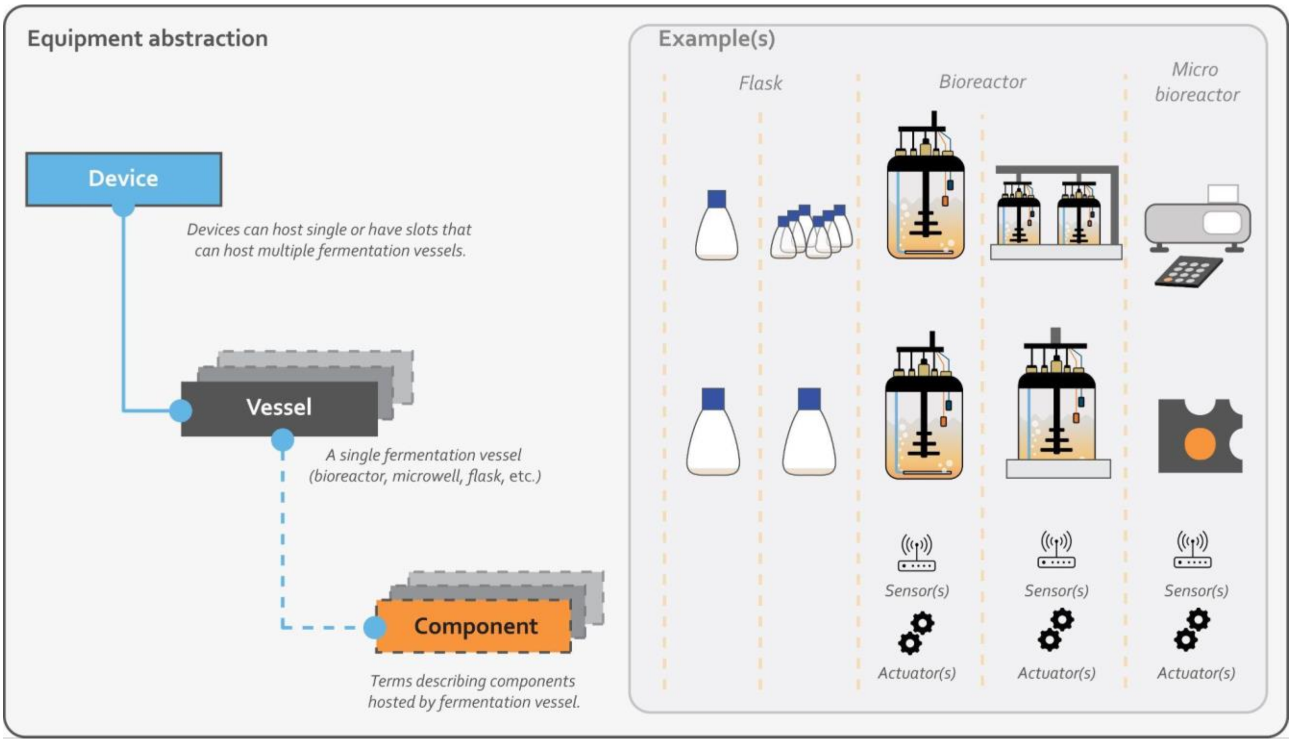

Figure 3. Overview of the MIFD metadata hierarchical structure. *The proposed hierarchy ensures the support for the majority of fermentation equipment configurations currently available in the IB field. Devices can contain a single* (i.e., *bioreactor*) *or multiple fermentation vessels* (i.e., *microwells*), *while each vessel can host multiple components* (i.e., *sensors and actuators*).

Ontology and FAIR Data Station

The concept of MIMs is tightly connected to efforts in enabling reproducible science, an aspect of science that has attracted significant attention during the last two decades[61]. However, to be

exploitable, structured metadata schemas such as MIMs must be incorporated into a usable format such as user-friendly software that researchers can use to organize their experiments, generated data, computational analyses, and/or any other effort to share with the scientific community. Furthermore, alongside being human readable, MIMs need to be resolvable by software systems in a form that is computationally tractable; that is, they must be unambiguous and contained within machine-readable formats. By standardising key experiment and equipment terms in both human and machine readable formats it will become not only far easier to reproduce or reuse existing datasets but allow for integrating data from multiple sources and automating data processing pipelines for data analytics. Facilitating advanced tasks like machine learning, digital twin simulations, and semantic querying over large datasets. In other words, consistent naming and structure are essential for any data-driven or AI-based workflow.

Therefore, to facilitate the programmatic use of the MIMs presented here, a LinkML schema encompassing the proposed terms for MIFE and MIFD was created. LinkML is not tied to a specific format or serialisation, thus it promotes interoperability across data formats and systems. Once defined, a LinkML schema can be transformed into multiple representations—including JSON Schema, OWL, SQL Database Schemas, and Python Data Classes. The LinkML representation was developed using a semi-automated process: an initial document was automatically generated, after parsing **Supplementary Files 1 and 2**, and was manually refined to remove extraneous artefacts. The resulting schema is available here: https://gitlab.com/bioindustry-4.0/mim_ontology. Because the ontology can be generated computationally, as new terms and relationships are required or the same terms and relationships evolve as needs change, the underlying ontology can be recreated with minimal manual input. The IB community is encouraged to submit requests for adding new or updating existing terms through the issue tracker of the gitlab repository.

In addition, researchers who wish to avail themselves of MIFE and MIFD can use FAIRDOM-SEEK[50] and FAIR DS which have been adapted to allow metadata to be defined in line with the MIMs presented here. FAIR DS is a tool that can generate metadata templates in the form of Excel workbooks and once filled in, validate the contents to ensure values that are entered by a researcher comply with the expected value. These templates enable researchers to systematically record experimental metadata in a FAIR and consistently, ensuring that metadata can be effectively shared, analyzed and reused across experiments within and between facilities[35].

Key features of the FAIR DS include:

1. Automated Metadata Structuring: The system standardizes metadata capture, ensuring compliance with FAIR principles.
2. Interoperability with FAIRDOM-SEEK: The templates facilitate direct integration with FAIRDOM-SEEK, streamlining (meta-)data sharing and collaboration.
3. Customizable Metadata Fields: Users can tailor the templates to accommodate domain-specific requirements while maintaining consistency across datasets.
4. Ontology Integration: The system leverages controlled vocabularies and ontologies to enhance data interoperability and semantic consistency.

To ensure data is findable, a key principle of FAIR, users can upload the validated metadata files from FAIR DS to FAIRDOM-SEEK. FAIRDOM-SEEK is an open-source web-based platform, that adheres to the FAIR principles[2], for cataloguing and sharing heterogeneous scientific research datasets, models or simulations, processes and research outcomes. It was initially introduced as a tool that facilitates the sharing and integration of data in the Systems Biology domain, however, it

was quickly adopted by other domains. To showcase the seamless and automated integration of the work presented here within FAIRDOM-SEEK and similar research data management platforms, we integrated MIFE in FAIR DS[35]. Users can visit the FAIR DS web portal (accessible via https://fairds.fairbydesign.nl/) and consult the relevant tutorials to generate a (meta)data workbook template utilising MIFE, fill in experimental metadata, and then subsequently validate them with FAIR DS[35], and proceed with uploading them to relevant data management platforms (*e.g.*, IBISBA Knowledge Hub[62]). To demonstrate the usability of MIFE, Supplementary File 3 is a FAIR-DS-compliant MIFE template filled with metadata information. This file is based upon the dataset provided as part of the Industrial-scale penicillin simulation (IndPenSim)[63,64], as it provides a suitable use-case that would be the equivalent of large, industrial, scale fermentations (e.g., [23]).

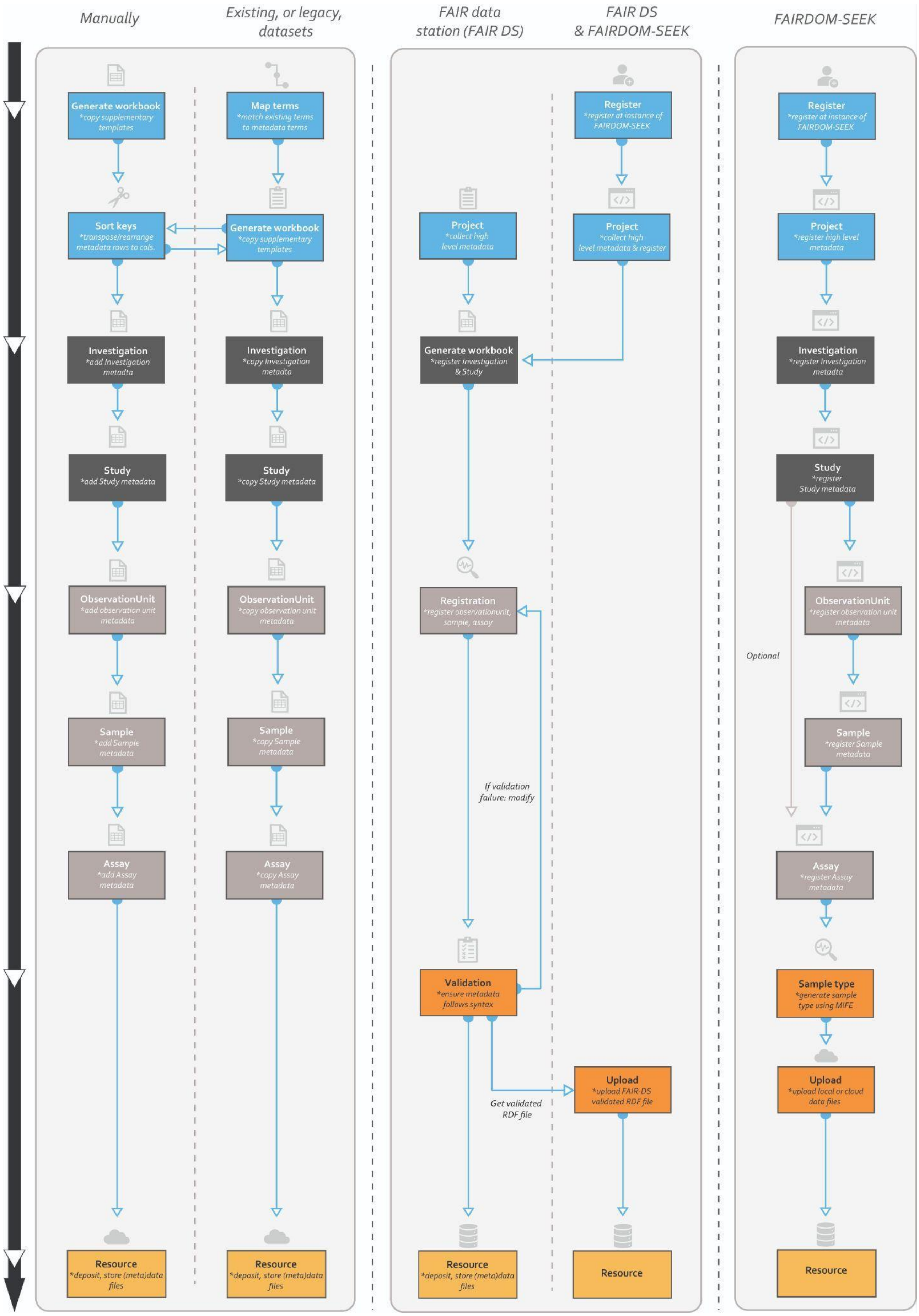

Figure 4. Flow diagram of how a user can utilise MIFE. *An individual can choose to manually create their metadata file or to utilise FAIR DS, FAIRDOM-SEEK, or a combination of the two.*

Provenance

In addition to providing robust metadata schemas for IB bioprocesses, this work aimed to design MIFE and MIFD in a manner that enables alignment with and seamless integrations into ongoing provenance standardization efforts. The Common Provenance Model (CPM)[9,26] is a data model for provenance representation based on PROV-DM, which serves as an open conceptual foundation for the *ISO 23494 Biotechnology – Provenance information model for biological material and data* series[9]. According to CPM, the most essential part of provenance is a *provenance backbone*, which is essentially a chain of standardized documentation of inputs (*backward connectors*) and outputs (*forward connectors*) of a documented process, also referred to as the *main activity*. Any other domain-specific information, including information about the sub-processes of the main activity, is appended to the backbone in a standardized way. As IB bioprocesses that are the main subject of the MIFE are typically only a single step in a broader workflow – starting from samples collection and processing, through a fermentation experiment, ending with the generated data processing and analysis – we see it important to be able to put the information from the experiment onto a standardized provenance chain. **Table 1** summarizes the elements of the MIFE and MIFD that we consider as basic building blocks to be integrated with the CPM, *i.e.* how the MIFE-related processes can correspond to the main activity in the CPM.

Table 1*. *Connecting MIFE and MIFD with the CPM. *List of MIFE terms representing a process that can be represented as the main activity of the CPM, and a list of its potential inputs and outputs that can be represented in the provenance backbone of the CPM. How specifically is this mapping implemented on the level of the CPM is dependent on the purpose of provenance collection (e.g., traceability, reusability or quality assessment, and reproducibility)*

CPM main activity	CPM Inputs (backward connectors)	CPM Outputs (forward connectors)
Observation unit	sample, vessel, protocol, feed materials, chemical compounds	data for analysis, (technical) logfiles
Sample processing	sample	processed sample(s)
Sample collection	source	sample

Conclusions

Since the early 21st century, the world has experienced an unprecedented rate of technological advancements, facilitating the constant emergence of breakthroughs across virtually every scientific discipline, in both academia and the private sector. Consequently, these technological advancements are fueling the Big Data era with an ever increasing stream of heterogeneous data that come in various, often not machine readable, forms. Voluminous data can provide the ideal substrate for artificial intelligence (AI) and explainable AI (XAI) approaches to grow and accelerate even further knowledge discovery and technological innovation.

However, access to unlimited data cannot on its own guarantee the development of trustworthy models, since frequently, the information describing the data, also known as metadata, has been repeatedly proven to be equally valuable if not more valuable than the actual data. MIMs and ontologies have been an integral part of data management and sharing as well as in facilitating interoperable science. Meticulously designed MIMs not only promote FAIRification in science and efficient business operation in the private sector, but they also empower AI to achieve its full potential.

As a field that has been the hotspot of intense multidisciplinary research with significant industrial applications, IB could not deviate from the aforementioned norm. The technological innovations have transformed the field with equipment that can record data of fermentation experiments at an unprecedented scale producing voluminous data in real-time. Understanding bioprocesses and developing accurate modeling procedures, that can facilitate informed decision making in IB, does not only require abundant and trustworthy data but also comprehensive data annotation in the form of rich metadata.

In this study, we propose a set of metadata schemas, MIFE and MIFD, specifically designed to promote FAIR principles in IB. Additionally, the structural dependencies between the subsets of terms of these MIMs and the integration with FAIR DS as well as the implemented ontology offer a novel approach for data standardization and annotation. Most terms were mapped to existing ontologies to facilitate interoperability with existing infrastructures and well-established standards while enabling easier adoption of the proposed MIMs by the community. The integration with FAIR DS specifically, can significantly enhance user-experience by automating (meta)data validation prior to converting them to machine readable formats and using them for downstream modeling tasks. Additionally, FAIR DS based validation ensures the incorporation of data and metadata into FAIR-inspired repositories, such as FAIRDOM-SEEK.

Even though the purpose of MIMs is to distill the necessary information for describing data to a minimal set of metadata, a different strategy was selected in this study. Both MIFE and MIFD include an extensive list of metadata terms that is divided into three main categories; i) mandatory, ii) recommended and iii) optional. The mandatory terms correspond to the minimum information required to describe fermentation data, while the recommended and optional terms allow for a more thorough type of management. Especially in the case of the observation unit subset of MIFE terms, an exhaustive list of terms is provided, most of which are optional. Filling a template with this many terms might seem daunting for day-to-day operations, hence the categorization into mandatory, recommended and optional terms. Although MIFE and MIFD contain a comprehensive set of metadata terms and they exhibit a generic hierarchical structure design that can extend their application beyond IB-related fermentations, there might be cases where these models prove insufficient. In simple terms, a MIM cannot include a term for every possible concept. The development of new MIMs and the extension of existing ones should become a community effort that will lead to the development of a robust foundation for open science and (meta)data standardization.

Despite the aforementioned limitations, the field of IB and any fermentation domain in general can significantly benefit from efforts that attempt to offer (meta)data standardization, such as MIFE and MIFD, in a multifaceted way. Transitioning from basic research to industrial scale applications can be accelerated and AI can be seamlessly integrated into every step of this process. Interoperable data accompanied with rich metadata can accelerate knowledge discovery and enable the exploitation of explainable AI methods to understand the complex underlying mechanisms of bioprocesses.

Availability of Source Code and Requirements

Python code for generating LinkML ontology schema, MIFE and MIFD ontologies in owl format: https://gitlab.com/bioindustry-4.0/mim_ontology

FAIR Data Station can be accessed: https://fairds.fairbydesign.nl/ with tutorials for validation: http://docs.fairbydesign.nl

Ontology repository service (MIFD): https://bioportal.bioontology.org/ontologies/MIFD

Ontology repository service (MIFE): https://bioportal.bioontology.org/ontologies/MIFE

The IBISBA Knowledge Hub (IBISBAkHub), an instance of FAIRDOM-SEEK, with MIFE integrated: https://ibisbahub.eu/

The metadata models presented in this study are published under the GNU General Public License (GPL) version 3.0.

Abbreviations

AI: artificial intelligence
BFO: basic formal ontology
ChEBI: chemical entities of biological interest
CPM: common provenance model
EBO: environmental biorefinery ontology
EFO: experimental factor ontology
ENVO: environment ontology
FAIR: findable, accessible, interoperable and reproducible
FAIR DS: FAIR data station
FOAF: friend-of-a-friend
IB: industrial biotechnology
ISA: investigation, study, assay
JERM: just enough results model
JSON: javascript object notation
KOS: knowledge organization systems
LinkML: linked data modeling language
MIAME: minimum information about a microarray experiment
MIFE: minimum information for fermentation experiments
MIFD: minimum information for fermentation devices
MIM: minimum information models
MIRIAM: minimum information requested in the annotation of biochemical models
MIxS: minimum information about any (x) sequence
ML: machine learning
OBI: Ontology for Biomedical Investigations
OBO: open biological and biomedical ontology
OESO: ontology of experimental scientific objects
OM: ontology of units of measure
ORCID: open researcher and contributor identifier
OWL: web ontology language
PO2: process and observation ontology
PROV-DM: PROV data model
PROV-O: PROV ontology
RDF: Resource Description Framework file format
SOSA: sensor, observation, sample, and actuator
SSN: semantic sensor network
SQL: structured query language
XAI: explainable AI

Competing Interests

The authors declare that they have no competing interests.

Funding

The authors disclose receipt of the following financial support for the research, authorship, and publication of this article: European Union's Horizon 2020 research and innovation programme projects 'RI services to promote deep digitalization of Industrial Biotechnology - towards smart biomanufacturing' (BIOINDUSTRY 4.0, grant agreement n° 101094287 [https://doi.org/10.3030/101094287]). J.J.K acknowledges the Dutch Research Council (NWO), and Wageningen University & Research for their financial contribution to the UNLOCK initiative (NWO: 184.035.007).

Authors' Contributions

GKG, BM, AB, MC, EF, JJK and SMAV developed MIFE and MIFD. PB, RW and AvG provided extensive feedback for refining MIFE and MIFD. MC, AB, EF, BM, JJK and GKG connected MIFE and MIFD terms to existing ontologies. GKG, BM, AB, MC, EF, JJK and RW wrote the manuscript. AvG and DCC provided extensive feedback for refining the manuscript. BM prepared all figures. MC developed the Python code for generating the LinkML ontology schema. HM, MW, CAAL, SO, MA and TD proofread the manuscript. GKG and JJK coordinated the study.

Acknowledgements

We thank the participants of the BIOINDUSTRY 4.0 project and associated BIOINDUSTRY 4.0 meetings for their discussions, attendance at workshops, and the insights provided that greatly improved MIFE and MIFD.

References

1. Pujol Priego, L., Wareham, J. & Romasanta, A. K. S. The puzzle of sharing scientific data. *Ind. Innov.* **29**, 219–250 (2022).
2. Wilkinson, M. D. *et al.* The FAIR Guiding Principles for scientific data management and stewardship. *Sci. Data* **3**, 160018 (2016).
3. EOSC Association. *EOSC Association* https://eosc.eu/.
4. Begley, C. G. & Ioannidis, J. P. A. Reproducibility in science. *Circ. Res.* **116**, 116–126 (2015).
5. Begley, C. G. & Ellis, L. M. Drug development: Raise standards for preclinical cancer research: Drug development. *Nature* **483**, 531–533 (2012).
6. Fung, A. W. S. Utilizing connectivity and data management system for effective quality management and regulatory compliance in point of care testing. *Pract. Lab. Med.* **22**, e00187 (2020).
7. Grønning, N. Data management in a regulatory context. *Front. Med. (Lausanne)* **4**, 114 (2017).
8. Plass, M. *et al.* Provenance of specimen and data - A prerequisite for AI development in computational pathology. *N. Biotechnol.* **78**, 22–28 (2023).
9. Wittner, R. *et al.* Toward a common standard for data and specimen provenance in life sciences. *Learn. Health Syst.* **8**, e10365 (2024).
10. Ancelin, M. *et al.* Addressing semantic ambiguity in biotechnology: Proposals from the European research infrastructure IBISBA. *N. Biotechnol.* **88**, 83–88 (2025).
11. Jonkers, L. *et al.* Community guidelines to increase the reusability of marine microfossil assemblage data. *J. Micropalaeontol.* **44**, 145–168 (2025).
12. Wieczorek, J. *et al.* Darwin Core: an evolving community-developed biodiversity data standard. *PLoS One* **7**, e29715 (2012).
13. Bubnicki, J. W. *et al.* Camtrap DP: an open standard for the FAIR exchange and archiving of camera trap data. *Remote Sens. Ecol. Conserv.* **10**, 283–295 (2024).
14. Scotson, L. *et al.* Best practices and software for the management and sharing of camera trap data for small and large scales studies. *Remote Sens. Ecol. Conserv.* **3**, 158–172 (2017).
15. Fegraus, E. H., Andelman, S., Jones, M. B. & Schildhauer, M. Maximizing the value of ecological data with structured metadata: An introduction to ecological metadata language (EML) and principles for metadata creation. *Bull. Ecol. Soc. Am.* **86**, 158–168 (2005).
16. Caufield, J. H. *et al.* A reference set of curated biomedical data and metadata from clinical case reports. *Sci. Data* **5**, 180258 (2018).
17. Field, D. *et al.* The minimum information about a genome sequence (MIGS) specification. *Nat. Biotechnol.* **26**, 541–547 (2008).
18. Yilmaz, P. *et al.* Minimum information about a marker gene sequence (MIMARKS) and minimum information about any (x) sequence (MIxS) specifications. *Nat. Biotechnol.* **29**, 415–420 (2011).
19. Brazma, A. *et al.* Minimum information about a microarray experiment (MIAME)-toward standards for microarray data. *Nat. Genet.* **29**, 365–371 (2001).
20. Le Novère, N. *et al.* Minimum information requested in the annotation of biochemical models (MIRIAM). *Nat. Biotechnol.* **23**, 1509–1515 (2005).
21. Fung Shek, C. & Betenbaugh, M. Taking the pulse of bioprocesses: at-line and in-line monitoring of mammalian cell cultures. *Curr. Opin. Biotechnol.* **71**, 191–197 (2021).
22. Castelvecchi, D. Can we open the black box of AI? *Nature* **538**, 20–23 (2016).
23. Metcalfe, B. *et al.* Towards a machine learning operations (MLOps) soft sensor for real-time predictions in industrial-scale fed-batch fermentation. *Comput. Chem. Eng.* **194**, 108991 (2025).
24. *Digital Twins.* (Springer Nature, Cham, Switzerland, 2021).
25. PROV-O: The PROV ontology. https://www.w3.org/TR/prov-o/.
26. Wittner, R. *et al.* Lightweight distributed provenance model for complex real-world environments. *Sci. Data* **9**, 503 (2022).
27. Second staff working document on data spaces. *Shaping Europe's digital future* https://digital-strategy.ec.europa.eu/en/library/second-staff-working-document-data-spaces.

28. Regulation - EU - 2024/1689 - EN - EUR-Lex. https://eur-lex.europa.eu/eli/reg/2024/1689/oj.
29. Janowicz, K., Haller, A., Cox, S. J. D., Le Phuoc, D. & Lefrançois, M. SOSA: A lightweight ontology for sensors, observations, samples, and actuators. *Web Semant.* **56**, 1–10 (2019).
30. Compton, M. *et al.* The SSN ontology of the W3C semantic sensor network incubator group. *Web Semant.* **17**, 25–32 (2012).
31. Kalemi, E. & Martiri, E. FOAF-academic ontology: A vocabulary for the academic community. in *2011 Third International Conference on Intelligent Networking and Collaborative Systems* (IEEE, 2011). doi:10.1109/incos.2011.94.
32. Spear, A. D., Ceusters, W. & Smith, B. Functions in Basic Formal Ontology. *Appl. Ontol.* **11**, 103–128 (2016).
33. Rocca-Serra, P. *et al.* Investigation-Study-Assay, a toolkit for standardizing data capture and sharing. in *Open Source Software in Life Science Research* 173–188 (Elsevier, 2012).
34. Wolstencroft, K. *et al.* Semantic data and models sharing in systems biology: The just enough results model and the SEEK platform. in *Advanced Information Systems Engineering* 212–227 (Springer Berlin Heidelberg, Berlin, Heidelberg, 2013).
35. Nijsse, B., Schaap, P. J. & Koehorst, J. J. FAIR data station for lightweight metadata management and validation of omics studies. *Gigascience* **12**, (2022).
36. Malone, J. *et al.* Modeling sample variables with an Experimental Factor Ontology. *Bioinformatics* **26**, 1112–1118 (2010).
37. *Process Observation Ontology Food Science PO2/TransformON, Ontology Data Integration Food, Feed*.
38. Oeso-core. https://agroportal.lirmm.fr/ontologies/OESO-CORE.
39. EBO. https://agroportal.lirmm.fr/ontologies/EBO.
40. Buttigieg, P. L. *et al.* The environment ontology in 2016: bridging domains with increased scope, semantic density, and interoperation. *J. Biomed. Semantics* **7**, 57 (2016).
41. Hastings, J. *et al.* The ChEBI reference database and ontology for biologically relevant chemistry: enhancements for 2013. *Nucleic Acids Res.* **41**, D456–63 (2013).
42. Walls, R. L. *et al.* Meeting report: advancing practical applications of biodiversity ontologies. *Stand. Genomic Sci.* **9**, 17 (2014).
43. Sumner, L. W. *et al.* Proposed minimum reporting standards for chemical analysis Chemical Analysis Working Group (CAWG) Metabolomics Standards Initiative (MSI). *Metabolomics* **3**, 211–221 (2007).
44. Jones, A. R. *et al.* Guidelines for reporting the use of column chromatography in proteomics. *Nat. Biotechnol.* **28**, 654 (2010).
45. Schober, I. *et al.* BacDive in 2025: the core database for prokaryotic strain data. *Nucleic Acids Res.* **53**, D748–D756 (2025).
46. Bandrowski, A. *et al.* The ontology for Biomedical Investigations. *PLoS One* **11**, e0154556 (2016).
47. Whetzel, P. L. *et al.* BioPortal: enhanced functionality via new Web services from the National Center for Biomedical Ontology to access and use ontologies in software applications. *Nucleic Acids Res.* **39**, W541–5 (2011).
48. Papoutsoglou, E. A. *et al.* Enabling reusability of plant phenomic datasets with MIAPPE 1.1. *New Phytol.* **227**, 260–273 (2020).
49. Wolstencroft, K. *et al.* The SEEK: a platform for sharing data and models in systems biology. *Methods Enzymol.* **500**, 629–655 (2011).
50. Wolstencroft, K. *et al.* SEEK: a systems biology data and model management platform. *BMC Syst. Biol.* **9**, 33 (2015).
51. Wolstencroft, K. *et al.* FAIRDOMHub: a repository and collaboration environment for sharing systems biology research. *Nucleic Acids Res.* **45**, D404–D407 (2017).
52. Rijgersberg, H., van Assem, M. & Top, J. Ontology of units of measure and related concepts. *Semantic Web* (2013) doi:10.3233/SW-2012-0069.
53. Weber, M. *et al.* PO2/TransformON, an ontology for data integration on food, feed, bioproducts and biowaste engineering. *Npj Sci. Food* **7**, 47 (2023).
54. Ontologies. *allotropefoundation* https://www.allotrope.org/ontologies.
55. Ison, J. *et al.* EDAM: an ontology of bioinformatics operations, types of data and identifiers, topics and formats. *Bioinformatics* **29**, 1325–1332 (2013).
56. Smith, B. *et al.* The OBO Foundry: coordinated evolution of ontologies to support biomedical

data integration. *Nat. Biotechnol.* **25**, 1251–1255 (2007).
57. Kaspersetz, L. *et al.* Management of experimental workflows in robotic cultivation platforms. *SLAS Technol.* **29**, 100214 (2024).
58. Frey, L. J. & Krull, R. Microbioreactors for process development and cell-based screening studies. *Adv. Biochem. Eng. Biotechnol.* **179**, 67–100 (2022).
59. EVS Explore. https://evsexplore.semantics.cancer.gov/evsexplore/welcome?terminology=ncit.
60. Mietzsch, E., Martini, D., Kolshus, K., Turbati, A. & Subirats, I. How agricultural digital innovation can benefit from semantics: The case of the AGROVOC multilingual thesaurus. *Eng. Proc.* **9**, 17 (2021).
61. Munafò, M. R. *et al.* A manifesto for reproducible science. in *Methodological issues and strategies in clinical research (5th ed.)* 947–967 (American Psychological Association, Washington, 2024).
62. IBISBA knowledge hub. https://hub.ibisba.eu/.
63. Goldrick, S., Ştefan, A., Lovett, D., Montague, G. & Lennox, B. The development of an industrial-scale fed-batch fermentation simulation. *J. Biotechnol.* **193**, 70–82 (2015).
64. Goldrick, S. *et al.* Modern day monitoring and control challenges outlined on an industrial-scale benchmark fermentation process. *Comput. Chem. Eng.* **130**, 106471 (2019).

Figure 1

Click here to access/download;Figure;Figure 1.jpg

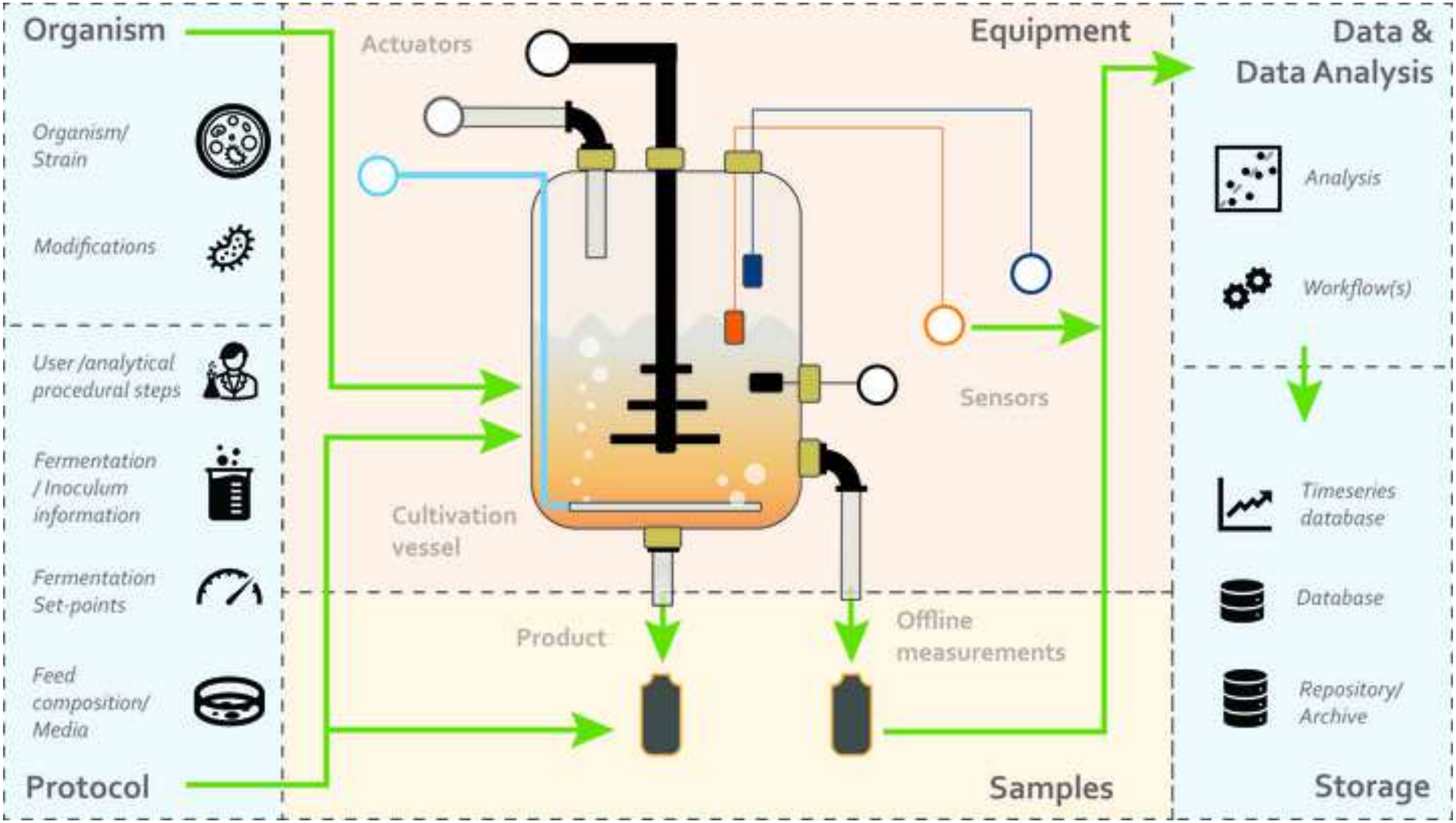

Figure 2

Click here to access/download;Figure;Figure 2.jpg

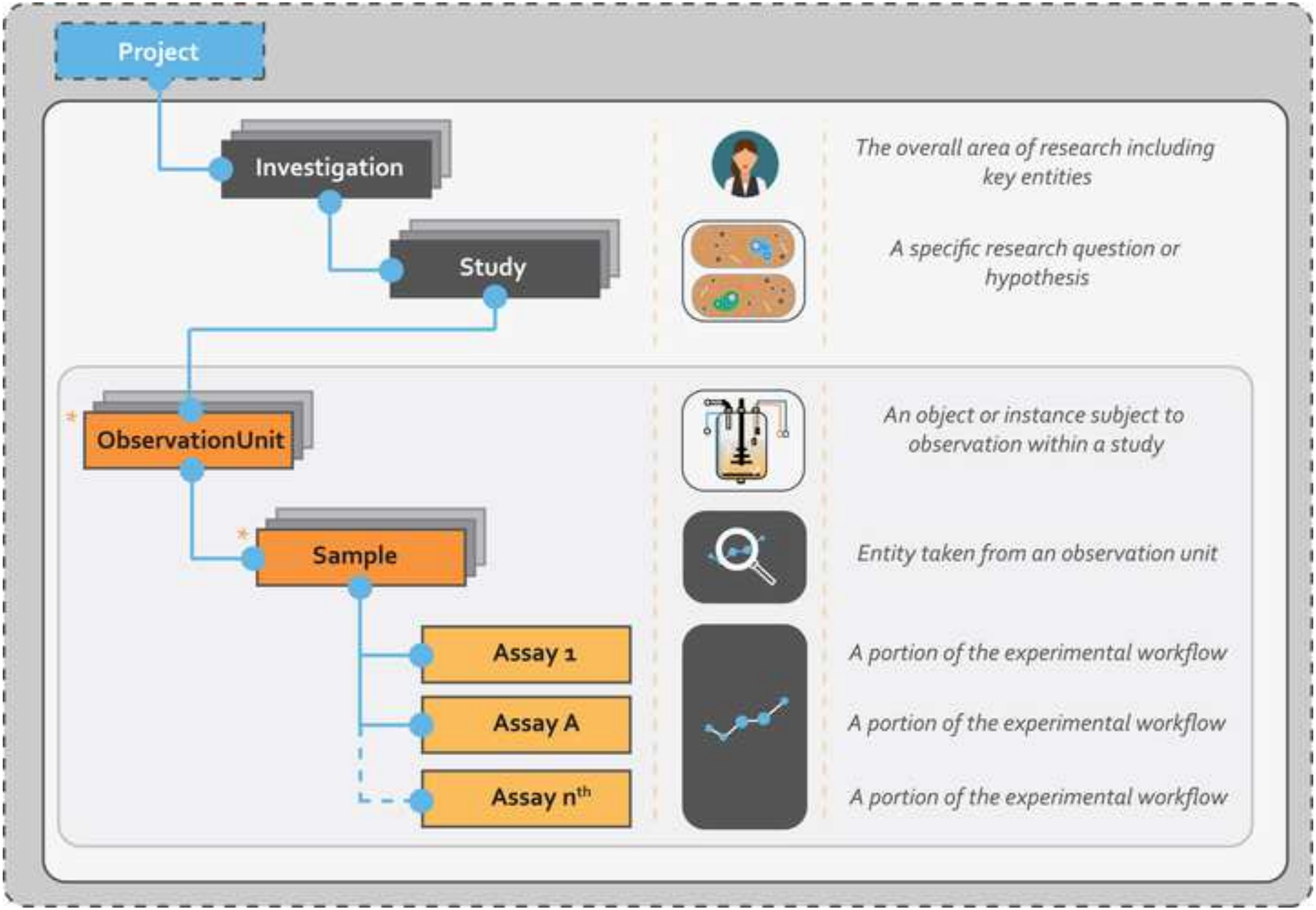

Figure 3

Click here to access/download;Figure;Figure 3.jpg

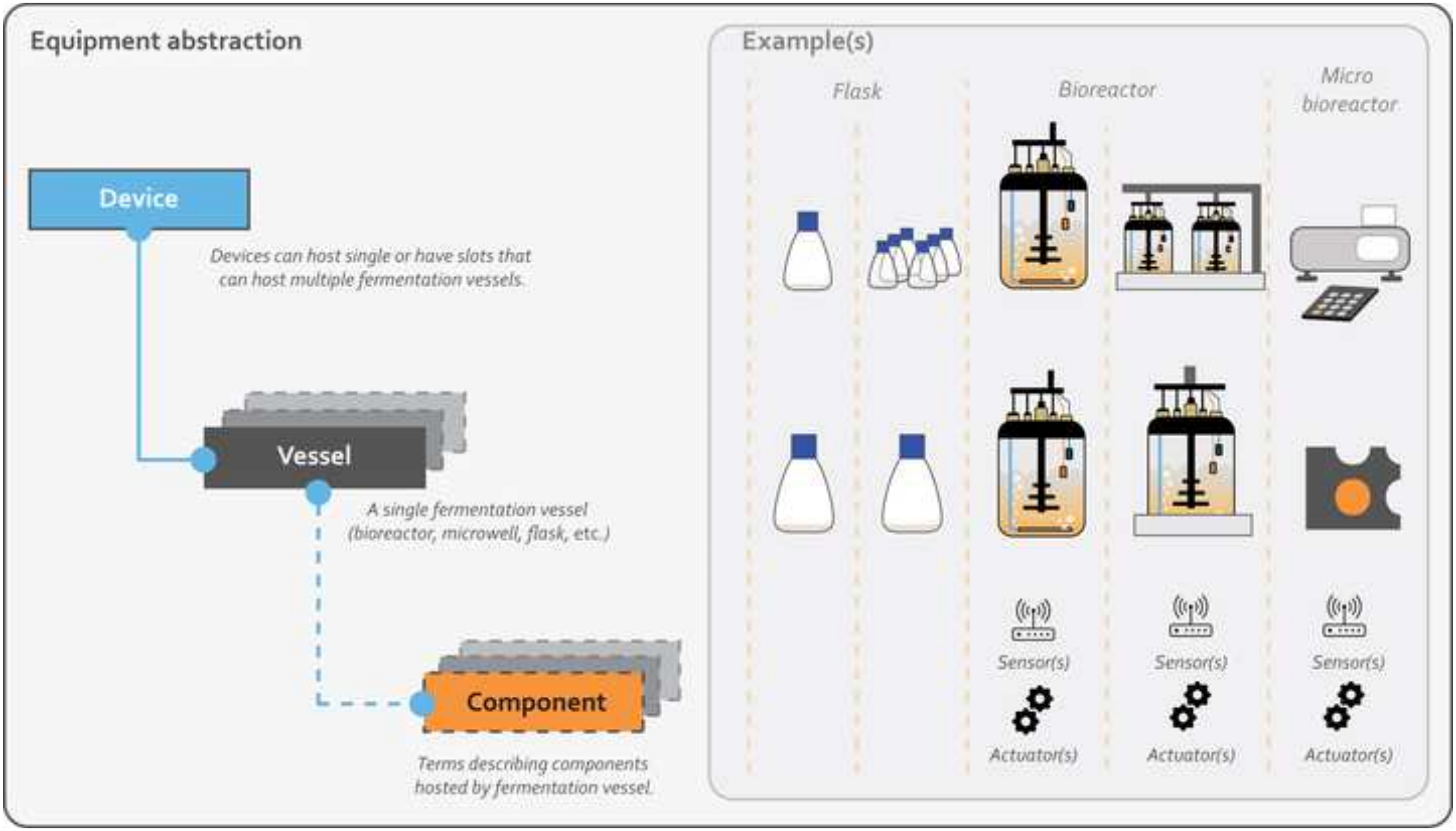

Figure 4

Click here to access/download;Figure;Figure 4.jpg

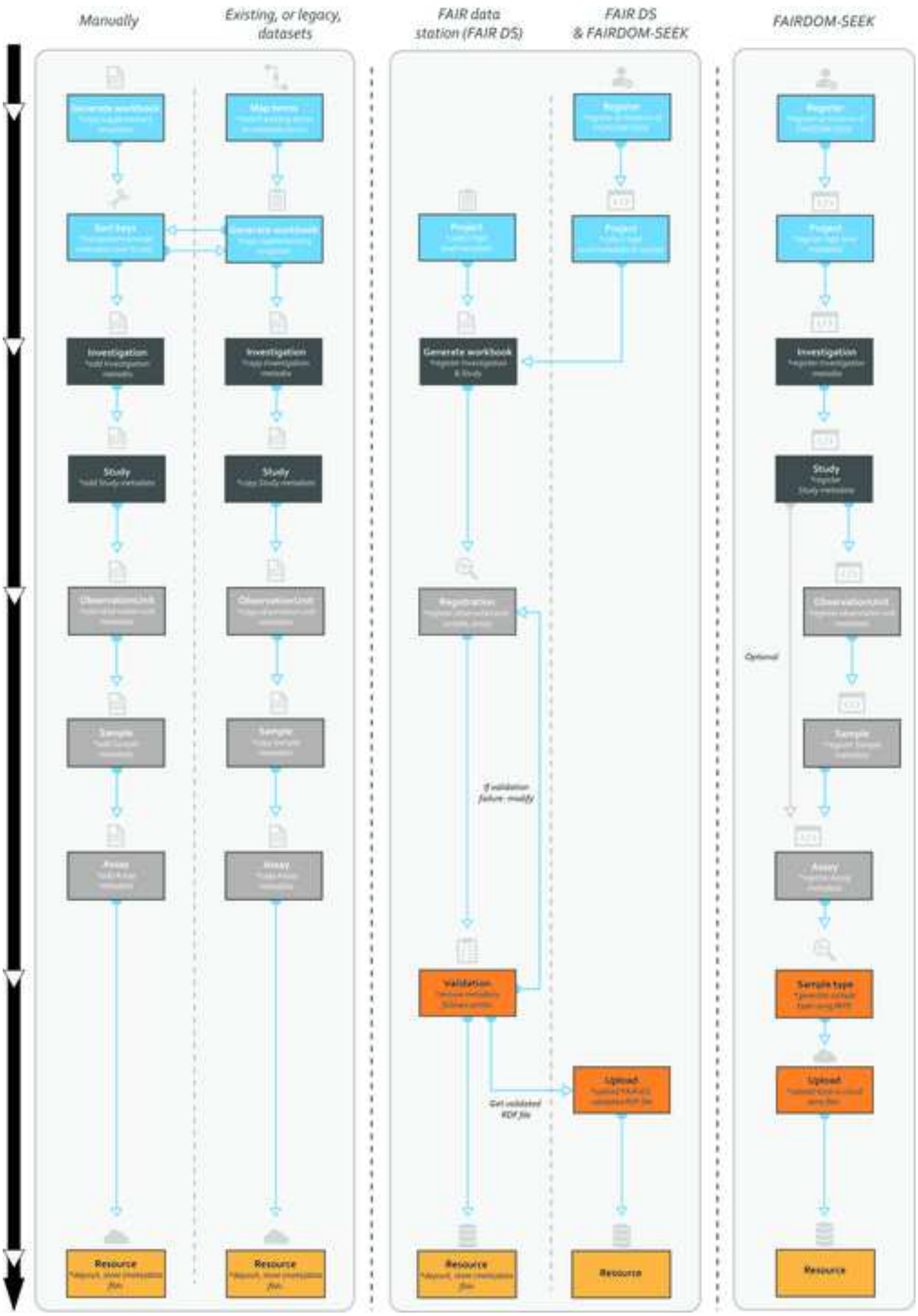

Supplementary File 1

Click here to access/download
Supplementary Material
Supplementary File 1.xlsx

Supplementary File 2

Click here to access/download
Supplementary Material
Supplementary File 2.xlsx

Click here to access/download
Supplementary Material
Supplementary File 3.xlsx

We would like to thank both of the reviewers for their invaluable suggestions and feedback on the original version of the manuscript. We believe that by addressing the comments, there was a significant improvement in how our work is presented.

The reviewers' comments can be found below in *italics* followed by a (detailed) response. Any adjustments on the manuscript have been colored in red.

Reviewer #1

The paper presents two complementary metadata schemas for describing fermentation experiments and the devices used in them. The topic is relevant and timely. As the authors argue, the definitions of commonly used metadata schemas align with the FAIR principles and contribute to community convergence. The importance of proper metadata, as defended by the authors, cannot be understated, which makes this paper particularly interesting.

However, I consider that this paper, in the current state, has two main issues: (1) the lack of test and validation and (2) contradiction on the purpose of providing a minimal information model.

Regarding 1, the authors presented motivation for such schemas, discussed related work and provided information about both MIFE and MIFD. However, there is no information about how the design considerations were defined and how the solution (the two metadata schemas) has been validated in real-world use cases and/or by the community. At least a test should have been described. As stated in the authors' submission instructions for GigaScience technical note papers (https://academic.oup.com/gigascience/pages/technical_note), "The tool or method needs to have been tested,...".

The information models presented in our study are part of the wider efforts of the BIOINDUSTRY4.0 consortium (https://www.bioindustry4.eu/) to advance the technological landscape of bioindustry in the EU. This consortium is composed of multiple institutes throughout Europe, bringing together highly interdisciplinary teams such as computer scientists, bioinformaticians, biotechnologists, biologists, and chemical engineers, among others.

MIFE and MIFD are the culmination of a two year effort of bringing this community together through workshops and webinars, reaching a consensus on the design requirements of structured metadata related to fermentation experiments. When writing the manuscript, we believed that the information about the aforementioned process would not provide any added value to the text, therefore we omitted it because it is irrelevant to the value and applicability of the models. However, if this is an important piece of information that should be in the manuscript, we could add it in a subsequent revision.

We agree with the reviewer that a usage example is currently missing from the manuscript. We added an additional supplementary file (Supplementary File 3) of a FAIR DS-ready template showcasing the usage of MIFE for sharing fermentation metadata. The file is ready to be validated by FAIR DS and then uploaded to FAIRDOM-SEEK (https://seek4science.org/) compliant platforms such as the IBISBA Knowledge Hub (https://hub.ibisba.eu/).

The second main issue is the term minimal information model, which is used in the title and throughout the paper. In my understanding, a minimal information model's purpose is to, as the term suggests, provide the minimal possible number of properties to describe a particular entity. And it seems that the authors agree with this understanding. In the conclusion section, the authors state that "Even though the purpose of MIMs is to distil the necessary information for describing data to a minimal set of metadata, a different strategy was

selected in this study.". Then why are the metadata schemas called Minimal Information for Fermentation and Devices? Using a MIM to thoroughly describe something seems like a contradiction. Certainly, if we only consider the mandatory properties, one could argue that they constitute a minimal information model. But then I don't think that the names of the schemas are appropriate, as only a small subset of them are, in fact, MIMs.

We would like to *thank the reviewer for the insightful comment. We agree that, conceptually, a Minimal Information Model (MIM) is meant to capture the essential set of metadata needed to describe a particular entity.*

However, in biotechnology, the range of entities and processes that can be described is extremely broad. The discipline spans molecular and cellular studies, enzyme and metabolic engineering, systems and synthetic biology, as well as fermentation technology, bioprocess and chemical engineering, catalysis, and eco-design.

Because of this diversity, what constitutes "minimal information" is highly context-dependent: the core metadata required to describe a microbial strain, for instance, differ fundamentally from those required to describe a fermentation process, a bioreactor, or an analytical device.

Our intention was therefore not to use the term MIM in its narrowest sense, but to extend its logic to multiple biotechnological contexts — each with its own "minimal" set of descriptors needed for reproducibility, interoperability, and data reuse. The terms Minimal Information for Fermentation (MIF) and Minimal Information for Devices (MID) thus reflect this adaptation: they preserve the MIM philosophy while acknowledging the multi-scale and multi-disciplinary nature of biotechnology.

Additionally, the development of MIFE and MIFD was initiated based on the need of standardizing fermentation metadata for data interoperability within the BIOINDUSTRY4.0 consortium, and due to the lack of IB-specific metadata standards in the literature. In early stages of the development both models included the metadata terms marked as mandatory, since the initial objective was to prepare a minimum set of terms for describing IB data. However, as the development was progressing and the BIOINDUSTRY4.0 consortium was exchanging opinions on data interoperability and modelling requirements through workshops and webinars, it became apparent that a richer and more flexible schema was required. To remain faithful to our initial objective of creating minimum information models for IB, we decided to divide terms into mandatory, recommended and optional.

Other minor comments include:

- In the "Minimum information models" section, the authors MIMs allow a community to adopt specific criteria deemed essential, required, or mandatory...". This is true of any data model, minimal or not. Almost every data modeling approach, e.g., UML class diagram, ER, etc., has a way of specifying cardinality where a minimal cardinally of 1 means mandatory and of 0 means optional.

The mandatory terms justify the reasoning behind the word minimum, while the remaining terms add the flexibility required by scientists involved in day-to-day research within the IB field. For instance, description of the purpose of the fermentation run is an essential descriptor for any fermentation process, but parameters such as what substance is the strain resistant to, or equipment used to feed the system may be context-dependent and therefore classified as recommended or optional.

- In section "Minimum information models in industrial biotechnology", the authors state "To make sense of IB (experimental) data and ensure that users of such data understand what will occur, is occurring, and has

occurred (Figure 1) necessitates that a data consumer is provided with:...". Is this necessity based on community consensus, expert opinion or some other requirement? And is it true for any application of IB data?

As mentioned in the previous comments, this study is an effort of the BIOINDUSTRY4.0 consortium to establish a set of metadata standards for fermentation data. Through workshops and webinars, scientists from different disciplines reached a consensus of how bioprocess metadata should be organized for streamlining downstream analyses, such as establishing kinetic, or genome-scale models, machine learning model training and soft-sensor applications for digital twins. The goal of IB data analytics is to provide a better understanding of the utilized microbial factories, leading to improved performance of bioprocesses and optimized product yield. This process is frequently hindered by the lack of metadata standards, presenting a significant obstacle to chemical engineers and computer scientists that attempt to develop predictive models and digital twin infrastructures.

- The title of the section "Overview of existing minimum information models" seems inadequate as it describes ontologies and vocabularies that are, arqueable, not minimal, e.g., BFO, SOSA, SSN, etc.

In this section we attempted to provide an overview of knowledge organization systems that are potentially relevant to IB but not specific to IB, at least to the extent that is required by the IB community. We agree with the reviewer that since these models are not minimum mode, the title of the section should be adjusted accordingly. Thus, we changed the title to "Overview of existing knowledge organization systems".

- In the sentence starting with "The application core Ontology of Experimental Scientific Objects (OESO-CORE) reuses...", what are the definitions of core and application ontologies? It doesn't seem that FOAF, SOSA and PROV-O are at the same level, and one could argue that, at least, FOAF and PROV-O are not upper-level ontologies. Maybe core ontologies.

We agree that SOSA etc. are not on the same level as BFO (upper-level), and are rather core ontologies. We have corrected the text in the article as follows: "It reuses concepts from upper-level and core ontologies (*e.g.*, BFO, SOSA)" and "The application core Ontology of Experimental Scientific Objects (OESO-CORE) reuses concepts from core ontologies (e.g., FOAF, SOSA, PROV-O)".

- Subsection "Ease-of-use": from the Gitlab files, it seems that the url column represents the object type or the datatype.

We would like to thank the reviewer for the apt remark. It was intended that the url column would be used for generating the linkML file. However, the name "url" does not indeed cover the heterogeneity of this column. To remain general, we renamed it as "object" (it refers to the triplet structure, where we have a subject, here corresponding to the column term, a predicate (column predicate), and an object (the url column that was renamed to object). The object column basically represents the value of the property: a resource or a literal (*i.e.*, a string, number, date)

Reviewer #2

The manuscript describes the development of two new standards, minimum information for fermentation experiments (MIFE) and devices (MIFD), addressing challenges in these communities to report data within a standardized framework.

Well written, sufficient justification provided in the background section.

-- when providing examples, remove 'etc

We removed 'etc from the relevant parts of the text.

-- Overview of existing minimum information models

In this section, I would have expected consideration of OBI: Ontology for Biomedical Investigations. Are there portions of OBI that would be pertinent to the developed standards?

We would like to thank the reviewer for the apt remark. Indeed, even though OBI is developed according to Biomedical standards, some individual concepts could be applicable to IB as well. At an earlier stage of MIFE and MIFD development, we considered using OBI terms related to, i.e., studies and investigations. However, we decided to include relevant terms from other metadata schemas, whose domain seemed closer to industrial biotechnology than the biomedical field. We have added relevant text mentioning OBI in the "Overview of existing knowledge organization systems" section.

-- Briefly outline methods utilized to identify/map individual terms within the standards to other standards, and how these mappings/re-use of terms were integrated.

To map the terms, we conducted research in various ontology portals, such as Bioportal and Agroportal. We considered broader and narrower concepts, as well as definitions. We used SKOS relationships (e.g., exact match) to integrate these mappings into the MIMs. These relationships are visible in the MIM publication on Bioportal, within each class.

It is noted in the Aims and Objective section:

Some metadata terms included in MIFD are mapped to existing ontologies.

-- How many terms, from which ontologies? What version of each ontology were the terms mapped to?

The ontology versions used were those from May 2025. Regarding the ontologies used for the mapping, they are mentioned in the manuscript in the sections "Minimum information for fermentation experiments" and "Minimum information for fermentation devices" (and references cited therein). In total, 89 terms in the MIFE and 26 in MIFD were mapped to other KOS concepts. We added relevant information in the two sections mentioned above.

--In the Design Considerations section, it is noted:

-- "what ontological class from an existing ontology it extends" and "the URL of a matching, existing, ontology concept ("url" column" --> Is the ID of the original term retained?

The classes of existing ontologies were mapped and not imported into the MIM, so the IDs of the original terms were not retained. MIFD and MIFE have their own ID system.

- Two edits for this sentence:

From: Minimum Information about any (X) Sequence (MIXS) and its subcategories from the Genomics Standards Consortium

to: Minimum Information about any (X) Sequence (MIxS) and its subcategories from the Genomic Standards Consortium

We made the proposed change into the relevant part of the text.

- Include the specific license associated with the standards.e.g., CCBY or CCO

-- Add this to the Availability statement.

We added a sentence specifying the licence associated to MIFE and MIFD in the "Availability of Source Code and Requirements" section.

-- when using e.g. -- always add a comma at the end, e.g.,

-- At the end of the Ontology and FAIR Data Station, there is a e.g. without the comma

We made the appropriate adjustments in the text.

-- Figure 4 - the text in the figure is blurry and the font size could be increased for readability

We reworked the font size in figure 4 to improve readability. We also provide all figures as separate files.

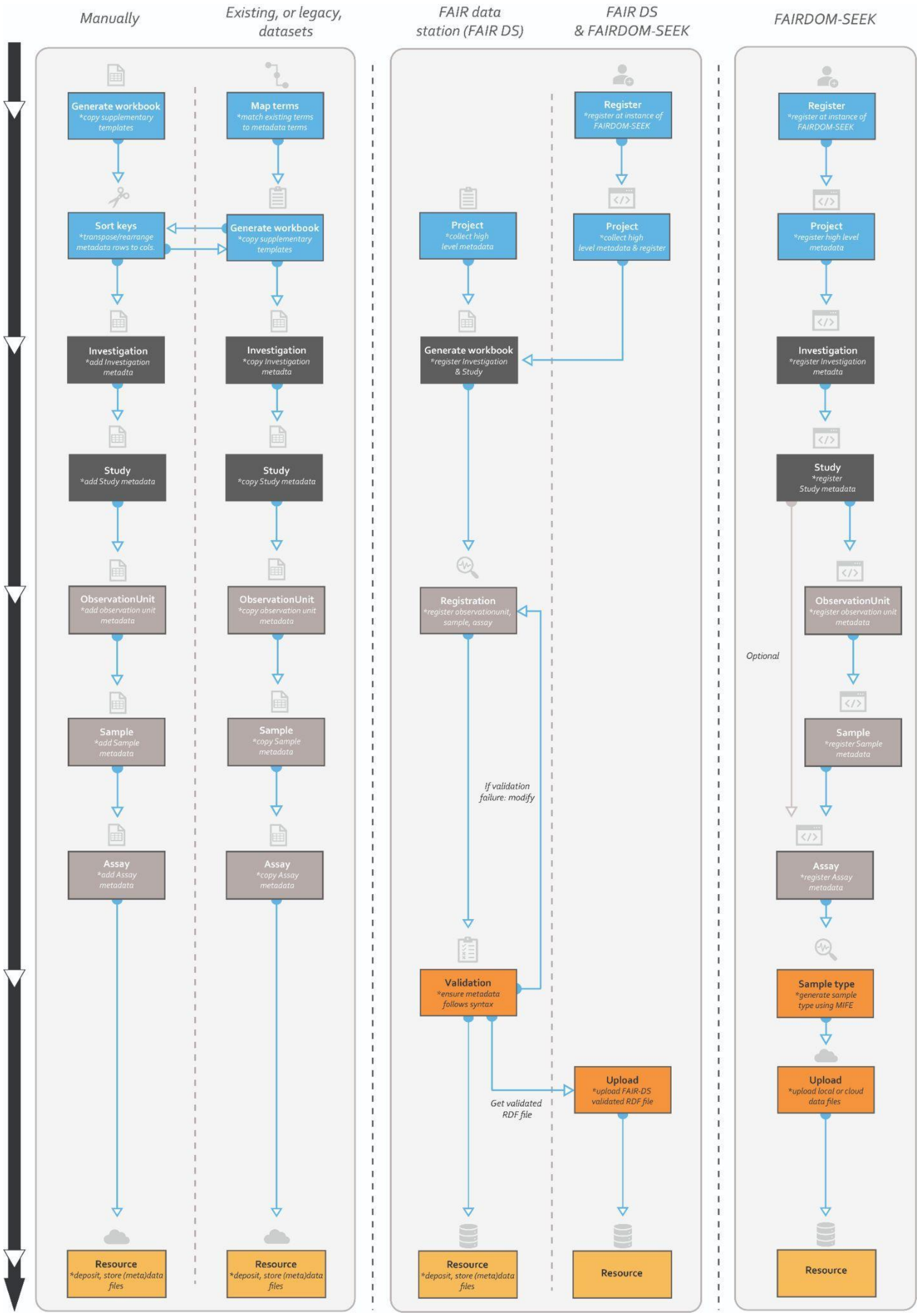

-- Conclusion

-- Is there a mention in the manuscript for gathering and integrating updates to the standards? How is this planned for ? Do you want users to submit issues to: https://gitlab.com/bioindustry-4.0/mim_ontology. Are

there datasets in preparation for this standard? Where will the datasets be submitted ? How often will updates to the schema be made available, that is, what is the planned update cycle?

We have included an additional supplementary file (Supplementary File 3) that includes a FAIR DS-ready template, filled with metadata from a published dataset. This file is ready to be validated using FAIR DS and then uploaded into FAIRDOM-SEEK-compliant platforms such as the IBISBA Knowledge Hub. The IBISBAKhub has implemented the MIFE under extended metadata so any user of IBISBA can already submit using this standard. We added relevant text in the "Ontology and FAIR Data Station" section.

Since the schema is an integral component of the IBISBA RI metadata standards, updates will happen based on the needs of IBISBA. Regardless, the community can submit requests about additions or adjustments to the schema through the GitLab issue tracking system. The consortium will be assessing such requests and evaluating their integration in future releases, if future releases are needed.
