# Supplementary material for: MIFE and MIFD: Minimum information for fermentation experiments and devices
Source: Gigascience. 2026 Apr 9;15:giag038. doi: 10.1093/gigascience/giag038 (PMC13184968; doi:10.1093/gigascience/giag038)
Supplement: giag038_Authors_Response_To_Reviewer_Comments_original_submission [file giag038_authors_response_to_reviewer_comments_original_submission.pdf]

Author's Response To Reviewer Comments

We would like to thank both of the reviewers for their invaluable suggestions and feedback on the original version of the manuscript. We believe that by addressing the comments, there was a significant improvement in how our work is presented.
The reviewers’ comments can be found below in italics followed by a (detailed) response. Any adjustments on the manuscript have been colored in red.
Reviewer #1
The paper presents two complementary metadata schemas for describing fermentation experiments and the devices used in them. The topic is relevant and timely. As the authors argue, the definitions of commonly used metadata schemas align with the FAIR principles and contribute to community convergence. The importance of proper metadata, as defended by the authors, cannot be understated, which makes this paper particularly interesting.
However, I consider that this paper, in the current state, has two main issues: (1) the lack of test and validation and (2) contradiction on the purpose of providing a minimal information model.
Regarding 1, the authors presented motivation for such schemas, discussed related work and provided information about both MIFE and MIFD. However, there is no information about how the design considerations were defined and how the solution (the two metadata schemas) has been validated in real-world use cases and/or by the community. At least a test should have been described. As stated in the authors' submission instructions for GigaScience technical note papers (https://academic.oup.com/gigascience/pages/technical_note), "The tool or method needs to have been tested,...".
The information models presented in our study are part of the wider efforts of the BIOINDUSTRY4.0 consortium (https://www.bioindustry4.eu/) to advance the technological landscape of bioindustry in the EU. This consortium is composed of multiple institutes throughout Europe, bringing together highly interdisciplinary teams such as computer scientists, bioinformaticians, biotechnologists, biologists, and chemical engineers, among others.
MIFE and MIFD are the culmination of a two year effort of bringing this community together through workshops and webinars, reaching a consensus on the design requirements of structured metadata related to fermentation experiments. When writing the manuscript, we believed that the information about the aforementioned process would not provide any added value to the text, therefore we omitted it because it is irrelevant to the value and applicability of the models. However, if this is an important piece of information that should be in the manuscript, we could add it in a subsequent revision.
We agree with the reviewer that a usage example is currently missing from the manuscript. We added an additional supplementary file (Supplementary File 3) of a FAIR DS-ready template showcasing the usage of MIFE for sharing fermentation metadata. The file is ready to be validated by FAIR DS and then uploaded to FAIRDOM-SEEK (https://seek4science.org/) compliant platforms such as the IBISBA Knowledge Hub (https://hub.ibisba.eu/).

The second main issue is the term minimal information model, which is used in the title and throughout the paper. In my understanding, a minimal information model's purpose is to, as the term suggests, provide the minimal possible number of properties to describe a particular entity. And it seems that the authors agree with this understanding. In the conclusion section, the authors state that "Even though the purpose of MIMs is to distil the necessary information for describing data to a minimal set of metadata, a different strategy was selected in this study.". Then why are the metadata schemas called Minimal Information for Fermentation and Devices? Using a MIM to thoroughly describe something seems like a contradiction. Certainly, if we only consider the mandatory properties, one could argue that they constitute a minimal information model. But then I don't think that the names of the schemas are appropriate, as only a small subset of them are, in fact, MIMs.
We would like to thank the reviewer for the insightful comment. We agree that, conceptually, a Minimal Information Model (MIM) is meant to capture the essential set of metadata needed to describe a particular entity.
However, in biotechnology, the range of entities and processes that can be described is extremely broad. The discipline spans molecular and cellular studies, enzyme and metabolic engineering, systems and synthetic biology, as well as fermentation technology, bioprocess and chemical engineering, catalysis, and

eco-design.
Because of this diversity, what constitutes “minimal information” is highly context-dependent: the core metadata required to describe a microbial strain, for instance, differ fundamentally from those required to describe a fermentation process, a bioreactor, or an analytical device.
Our intention was therefore not to use the term MIM in its narrowest sense, but to extend its logic to multiple biotechnological contexts — each with its own “minimal” set of descriptors needed for reproducibility, interoperability, and data reuse. The terms Minimal Information for Fermentation (MIF) and Minimal Information for Devices (MID) thus reflect this adaptation: they preserve the MIM philosophy while acknowledging the multi-scale and multi-disciplinary nature of biotechnology.
Additionally, the development of MIFE and MIFD was initiated based on the need of standardizing fermentation metadata for data interoperability within the BIOINDUSTRY4.0 consortium, and due to the lack of IB-specific metadata standards in the literature. In early stages of the development both models included the metadata terms marked as mandatory, since the initial objective was to prepare a minimum set of terms for describing IB data. However, as the development was progressing and the BIOINDUSTRY4.0 consortium was exchanging opinions on data interoperability and modelling requirements through workshops and webinars, it became apparent that a richer and more flexible schema was required. To remain faithful to our initial objective of creating minimum information models for IB, we decided to divide terms into mandatory, recommended and optional.

Other minor comments include:
- In the "Minimum information models" section, the authors MIMs allow a community to adopt specific criteria deemed essential, required, or mandatory...". This is true of any data model, minimal or not. Almost every data modeling approach, e.g., UML class diagram, ER, etc., has a way of specifying cardinality where a minimal cardinally of 1 means mandatory and of 0 means optional.
The mandatory terms justify the reasoning behind the word minimum, while the remaining terms add the flexibility required by scientists involved in day-to-day research within the IB field. For instance, description of the purpose of the fermentation run is an essential descriptor for any fermentation process, but parameters such as what substance is the strain resistant to, or equipment used to feed the system may be context-dependent and therefore classified as recommended or optional.

- In section "Minimum information models in industrial biotechnology", the authors state "To make sense of IB (experimental) data and ensure that users of such data understand what will occur, is occurring, and has occurred (Figure 1) necessitates that a data consumer is provided with:...". Is this necessity based on community consensus, expert opinion or some other requirement? And is it true for any application of IB data?
As mentioned in the previous comments, this study is an effort of the BIOINDUSTRY4.0 consortium to establish a set of metadata standards for fermentation data. Through workshops and webinars, scientists from different disciplines reached a consensus of how bioprocess metadata should be organized for streamlining downstream analyses, such as establishing kinetic, or genome-scale models, machine learning model training and soft-sensor applications for digital twins. The goal of IB data analytics is to provide a better understanding of the utilized microbial factories, leading to improved performance of bioprocesses and optimized product yield. This process is frequently hindered by the lack of metadata standards, presenting a significant obstacle to chemical engineers and computer scientists that attempt to develop predictive models and digital twin infrastructures.

- The title of the section "Overview of existing minimum information models" seems inadequate as it describes ontologies and vocabularies that are, arqueable, not minimal, e.g., BFO, SOSA, SSN, etc.
In this section we attempted to provide an overview of knowledge organization systems that are potentially relevant to IB but not specific to IB, at least to the extent that is required by the IB community. We agree with the reviewer that since these models are not minimum mode, the title of the section should be adjusted accordingly. Thus, we changed the title to “Overview of existing knowledge organization systems”.

- In the sentence starting with "The application core Ontology of Experimental Scientific Objects (OESO-CORE) reuses...", what are the definitions of core and application ontologies? It doesn't seem that FOAF, SOSA and PROV-O are at the same level, and one could argue that, at least, FOAF and PROV-O are not upper-level ontologies. Maybe core ontologies.

We agree that SOSA etc. are not on the same level as BFO (upper-level), and are rather core ontologies. We have corrected the text in the article as follows: “It reuses concepts from upper-level and core ontologies (e.g., BFO, SOSA)” and “The application core Ontology of Experimental Scientific Objects (OESO-CORE) reuses concepts from core ontologies (e.g., FOAF, SOSA, PROV-O)”.

- Subsection "Ease-of-use": from the Gitlab files, it seems that the url column represents the object type or the datatype.
We would like to thank the reviewer for the apt remark. It was intended that the url column would be used for generating the linkML file. However, the name "url" does not indeed cover the heterogeneity of this column. To remain general, we renamed it as "object" (it refers to the triplet structure, where we have a subject, here corresponding to the column term, a predicate (column predicate), and an object (the url column that was renamed to object). The object column basically represents the value of the property: a resource or a literal (i.e., a string, number, date)

Reviewer #2
The manuscript describes the development of two new standards, minimum information for fermentation experiments (MIFE) and devices (MIFD), addressing challenges in these communities to report data within a standardized framework.
Well written, sufficient justification provided in the background section.
-- when providing examples, remove 'etc
We removed ‘etc from the relevant parts of the text.

-- Overview of existing minimum information models
In this section, I would have expected consideration of OBI: Ontology for Biomedical Investigations. Are there portions of OBI that would be pertinent to the developed standards?
We would like to thank the reviewer for the apt remark. Indeed, even though OBI is developed according to Biomedical standards, some individual concepts could be applicable to IB as well. At an earlier stage of MIFE and MIFD development, we considered using OBI terms related to, i.e., studies and investigations. However, we decided to include relevant terms from other metadata schemas, whose domain seemed closer to industrial biotechnology than the biomedical field. We have added relevant text mentioning OBI in the “Overview of existing knowledge organization systems” section.

-- Briefly outline methods utilized to identify/map individual terms within the standards to other standards, and how these mappings/re-use of terms were integrated.
To map the terms, we conducted research in various ontology portals, such as Bioportal and Agroportal. We considered broader and narrower concepts, as well as definitions. We used SKOS relationships (e.g., exact match) to integrate these mappings into the MIMs. These relationships are visible in the MIM publication on Bioportal, within each class.

It is noted in the Aims and Objective section:
Some metadata terms included in MIFD are mapped to existing ontologies.
-- How many terms, from which ontologies? What version of each ontology were the terms mapped to?
The ontology versions used were those from May 2025. Regarding the ontologies used for the mapping, they are mentioned in the manuscript in the sections “Minimum information for fermentation experiments” and “Minimum information for fermentation devices” (and references cited therein). In total, 89 terms in the MIFE and 26 in MIFD were mapped to other KOS concepts. We added relevant information in the two sections mentioned above.

--In the Design Considerations section, it is noted:
-- "what ontological class from an existing ontology it extends" and "the URL of a matching, existing, ontology concept ("url" column" --> Is the ID of the original term retained?
The classes of existing ontologies were mapped and not imported into the MIM, so the IDs of the original terms were not retained. MIFD and MIFE have their own ID system.

- Two edits for this sentence:
From: Minimum Information about any (X) Sequence (MIXS) and its subcategories from the Genomics Standards Consortium

to: Minimum Information about any (X) Sequence (MIxS) and its subcategories from the Genomic Standards Consortium
We made the proposed change into the relevant part of the text.

- Include the specific license associated with the standards.e.g., CCBY or CCO
-- Add this to the Availability statement.
We added a sentence specifying the licence associated to MIFE and MIFD in the “Availability of Source Code and Requirements” section.

-- when using e.g. -- always add a comma at the end, e.g.,
-- At the end of the Ontology and FAIR Data Station, there is a e.g. without the comma
We made the appropriate adjustments in the text.

-- Figure 4 - the text in the figure is blurry and the font size could be increased for readability
We reworked the font size in figure 4 to improve readability. We also provide all figures as separate files.

-- Conclusion
-- Is there a mention in the manuscript for gathering and integrating updates to the standards? How is this planned for ? Do you want users to submit issues to: https://gitlab.com/bioindustry-4.0/mim_ontology. Are there datasets in preparation for this standard? Where will the datasets be submitted ? How often will updates to the schema be made available, that is, what is the planned update cycle?

We have included an additional supplementary file (Supplementary File 3) that includes a FAIR DS-ready template, filled with metadata from a published dataset. This file is ready to be validated using FAIR DS and then uploaded into FAIRDOM-SEEK-compliant platforms such as the IBISBA Knowledge Hub. The IBISBAKhub has implemented the MIFE under extended metadata so any user of IBISBA can already submit using this standard. We added relevant text in the “Ontology and FAIR Data Station” section. Since the schema is an integral component of the IBISBA RI metadata standards, updates will happen based on the needs of IBISBA. Regardless, the community can submit requests about additions or adjustments to the schema through the GitLab issue tracking system. The consortium will be assessing such requests and evaluating their integration in future releases, if future releases are needed.

em Editorial Manager®

Cookie Preference Center

We use cookies which are necessary to make our site work. We may also use additional cookies to analyze, improve and personalize our content and your digital experience. For more information, see our Cookie Policy.

You may choose not to allow some types of cookies. However, blocking some types may impact your experience of our site and the services we are able to offer. See the different category headings below to find out more or change your settings.

Allow all

Manage Consent Preferences

Strictly Necessary Cookies

Always active

These cookies are necessary for the website to function and cannot be switched off in our systems. They are usually only set in response to actions made by you which amount to a request for services, such as setting your privacy preferences, logging in or filling in forms. You can set your browser to block or alert you about these cookies, but some parts of the site will not then work.

Functional Cookies

These cookies enable the website to provide enhanced functionality and personalisation. They may be set by us or by third party providers whose services we have added to our pages. If you do not allow these cookies then some or all of these services may not function properly.

Performance Cookies

These cookies allow us to count visits and traffic sources so we can measure and improve the performance of our site. They help us to know which pages are the most and least popular and see how visitors move around the site.

Targeting Cookies

These cookies may be set through our site by our advertising partners. They may be used by those companies to build a profile of your interests and show you relevant adverts on other sites. If you do not allow these cookies, you will experience less targeted advertising.

Cookie List

Clear

Apply Cancel

Consent Leg.Interest

Confirm my choices

Powe
