# Supplementary material for: MIFE and MIFD: Minimum information for fermentation experiments and devices
Source: Gigascience. 2026 Apr 9;15:giag038. doi: 10.1093/gigascience/giag038 (PMC13184968; doi:10.1093/gigascience/giag038)
Supplement: giag038_Authors_Response_To_Reviewer_Comments_revision_1 [file giag038_authors_response_to_reviewer_comments_revision_1.pdf]

Author's Response To Reviewer Comments

We would like to thank both the reviewer and the editor for their suggestions. The comments can be found below in italics followed by the response. Any adjustments on the manuscript have been colored in red.
Reviewer #2
Examining the revised manuscript, I find that the revision does not adequately address reviewer #2's comments.
Unfortunately the reviewer has not specified which of the comments of reviewer 2 they do not believe we have addressed adequately so we are unsure how to address this point. Assuming we are referring to the same review (beginning with "The manuscript describes the development of two new standards, ..." in our previous submission we modified the text, added consideration of OBI, defined the number of mappings, and reformatted the figure according to Reviewer 2's helpful suggestions.

The background for the project development should be included, as should the testing. And not just in a supplemental file.
Background has been added to the aims and objectives section.

It is clear that this standard is not a 'Minimal Information Model', thus the title of the manuscript and usage of the 'minimal' modeling needs to be revised. , e.g., a 'Maximal-Minimal information model' To name this work as a minimal standard is misleading. The manuscript would benefit from a background section on existing minimal information models. Identifying other models that needed to be expanded beyond 'minimal', would strengthen the manuscript.
It is not misleading to use the term minimum information model as the three requirements (mandatory, optional, recommended) have, in some form, been a part of MIMs since their inception (~2000's). For instance, the 2008 paper MIFlowCyt: The minimum information about a flow cytometry experiment states the following: "The word shall is used to indicate that a particular item is an absolute requirement of the specification. It identifies information that is absolutely crucial in order to allow for interpretation of the experiment. Shall combined with if relevant indicates information that is not generally applicable but could impact the interpretation of a particular experiment in certain cases. The word should is used to indicate that a particular item is recommended but not required."
One could argue that taken as a single set, irrespective of whether they are mandatory or optional, they are a minimum set of requirements. Or one could argue that these represent the minimum mandatory, minimum recommended, and minimum optional sets of metadata. However, the reason for the convention of having mandatory, optional, and recommended requirements is simply pragmatism. Different researchers will disagree on what is needed or may not collect or have certain information (e.g., ORCIDs are not mandatory in science) and if we opted for making everything mandatory users would not want, or be able to use such criteria. It is therefore a trade-off between what would be ideal and what is possible.

Editor Comments:
GigaScience has also published a number of relevant papers on metadata standardization, FAIR data practices, and reproducible workflows in biotechnology that may help contextualize your work. We have listed several recent examples below for your reference. Citing some of these articles—where appropriate in the Background or Discussion—would help frame your contribution within ongoing community efforts and strengthen the presentation of related tools and frameworks.
1. Soto, J.M., Thomer, A., Alquaddoomi, F. et al. PEPhub: a database, web interface, and API for editing, sharing, and validating biological sample metadata. GigaScience 13, giae033 (2024).
https://doi.org/10.1093/gigascience/giae033
2. Arnaud, E., Alaux, M., Durand, S. et al. An ecosystem for producing and sharing metadata within the web of FAIR Data. GigaScience 14, giae111 (2025). https://doi.org/10.1093/gigascience/giae111
3. Nayfach, S., Zhu, Q., Eloe-Fadrosh, E.A. et al. MISIP: a data standard for the reuse and reproducibility of any stable isotope probing-derived nucleic acid sequence and experiment. GigaScience 13, giae071 (2024).
https://doi.org/10.1093/gigascience/giae071
4. Köster, J., Rasche, H., Grüning, B. et al. FAIR data retrieval for sensitive clinical research data in Galaxy.

GigaScience 13, giad099 (2024). https://doi.org/10.1093/gigascience/giad099
5. Droste, J., Laczny, C.C., Wilmes, P. et al. PathoGFAIR: a collection of FAIR and adaptable (meta)genomics workflows for (foodborne) pathogens detection and tracking. GigaScience 14, giae017 (2025).
https://doi.org/10.1093/gigascience/giaf017

We thank the editor for these suggestions and have added: https://doi.org/10.1093/gigascience/giae071; To the manuscript.

Editorial Manager®

Cookie Preference Center

We use cookies which are necessary to make our site work. We may also use additional cookies to analyze, improve and personalize our content and your digital experience. For more information, see our Cookie Policy.

You may choose not to allow some types of cookies. However, blocking some types may impact your experience of our site and the services we are able to offer. See the different category headings below to find out more or change your settings.

Allow all

Manage Consent Preferences

Strictly Necessary Cookies

Always active

These cookies are necessary for the website to function and cannot be switched off in our systems. They are usually only set in response to actions made by you which amount to a request for services, such as setting your privacy preferences, logging in or filling in forms. You can set your browser to block or alert you about these cookies, but some parts of the site will not then work.

Functional Cookies

These cookies enable the website to provide enhanced functionality and personalisation. They may be set by us or by third party providers whose services we have added to our pages. If you do not allow these cookies then some or all of these services may not function properly.

Performance Cookies

These cookies allow us to count visits and traffic sources so we can measure and improve the performance of our site. They help us to know which pages are the most and least popular and see how visitors move around the site.

Targeting Cookies

These cookies may be set through our site by our advertising partners. They may be used by those companies to build a profile of your interests and show you relevant adverts on other sites. If you do not allow these cookies, you will experience less targeted advertising.

Cookie List

Clear

Apply Cancel
Consent Leg.Interest

Confirm my choices

Power
