# Supplementary material for: MIFE and MIFD: Minimum information for fermentation experiments and devices
Source: Gigascience. 2026 Apr 9;15:giag038. doi: 10.1093/gigascience/giag038 (PMC13184968; doi:10.1093/gigascience/giag038)
Supplement: giag038_Reviewer_1_Report_original_submission — Reviewer 1 -- 7/15/2025 [file giag038_reviewer_1_report_original_submission.pdf]

Reviewer Report

Title: MIFE and MIFD: Minimum information for fermentation experiments and devices

Version: Original Submission **Date:** 7/15/2025

Reviewer name: Reviewer 1

Reviewer Comments to Author:

The paper presents two complementary metadata schemas for describing fermentation experiments and the devices used in them. The topic is relevant and timely. As the authors argue, the definitions of commonly used metadata schemas align with the FAIR principles and contribute to community convergence. The importance of proper metadata, as defended by the authors, cannot be understated, which makes this paper particularly interesting.

However, I consider that this paper, in the current state, has two main issues: (1) the lack of test and validation and (2) contradiction on the purpose of providing a minimal information model.

Regarding 1, the authors presented motivation for such schemas, discussed related work and provided information about both MIFE and MIFD. However, there is no information about how the design considerations were defined and how the solution (the two metadata schemas) has been validated in real-world use cases and/or by the community. At least a test should have been described. As stated in the authors' submission instructions for GigaScience technical note papers (https://academic.oup.com/gigascience/pages/technical_note), "The tool or method needs to have been tested,...".

The second main issue is the term minimal information model, which is used in the title and throughout the paper. In my understanding, a minimal information model's purpose is to, as the term suggests, provide the minimal possible number of properties to describe a particular entity. And it seems that the authors agree with this understanding. In the conclusion section, the authors state that "Even though the purpose of MIMs is to distil the necessary information for describing data to a minimal set of metadata, a different strategy was selected in this study.". Then why are the metadata schemas called Minimal Information for Fermentation and Devices? Using a MIM to thoroughly describe something seems like a contradiction. Certainly, if we only consider the mandatory properties, one could argue that they constitute a minimal information model. But then I don't think that the names of the schemas are appropriate, as only a small subset of them are, in fact, MIMs.

Other minor comments include:

- In the "Minimum information models" section, the authors MIMs allow a community to adopt specific criteria deemed essential, required, or mandatory...". This is true of any data model, minimal or not. Almost every data modeling approach, e.g., UML class diagram, ER, etc., has a way of specifying cardinality where a minimal cardinally of 1 means mandatory and of 0 means optional.
- In section "Minimum information models in industrial biotechnology", the authors state "To make sense of IB (experimental) data and ensure that users of such data understand what will occur, is occurring, and has occurred (Figure 1) necessitates that a data consumer is provided with:...". Is this necessity based on community consensus, expert opinion or some other requirement? And is it true for

any application of IB data?
- The title of the section "Overview of existing minimum information models" seems inadequate as it describes ontologies and vocabularies that are, arqueable, not minimal, e.g., BFO, SOSA, SSN, etc.
- In the sentence starting with "The application core Ontology of Experimental Scientific Objects (OESO-CORE) reuses...", what are the definitions of core and application ontologies? It doesn't seem that FOAF, SOSA and PROV-O are at the same level, and one could argue that, at least, FOAF and PROV-O are not upper-level ontologies. Maybe core ontologies.
- Subsection "Ease-of-use": from the Gitlab files, it seems that the url column represents the object type or the datatype.

Methods

Are the methods appropriate to the aims of the study, are they well described, and are necessary controls included? Choose an item.

Conclusions

Are the conclusions adequately supported by the data shown? Choose an item.

Reporting Standards

Does the manuscript adhere to the journal's guidelines on [minimum standards of reporting?] Choose an item.

Choose an item.

Statistics

Are you able to assess all statistics in the manuscript, including the appropriateness of statistical tests used? Choose an item.

Quality of Written English

Please indicate the quality of language in the manuscript: Choose an item.

Declaration of Competing Interests

Please complete a declaration of competing interests, considering the following questions:

- Have you in the past five years received reimbursements, fees, funding, or salary from an organisation that may in any way gain or lose financially from the publication of this manuscript, either now or in the future?
- Do you hold any stocks or shares in an organisation that may in any way gain or lose financially from the publication of this manuscript, either now or in the future?

- Do you hold or are you currently applying for any patents relating to the content of the manuscript?
- Have you received reimbursements, fees, funding, or salary from an organization that holds or has applied for patents relating to the content of the manuscript?
- Do you have any other financial competing interests?
- Do you have any non-financial competing interests in relation to this paper?

If you can answer no to all of the above, write 'I declare that I have no competing interests' below. If your reply is yes to any, please give details below.

I declare that I have no competing interests.

I agree to the open peer review policy of the journal. I understand that my name will be included on my report to the authors and, if the manuscript is accepted for publication, my named report including any attachments I upload will be posted on the website along with the authors' responses. I agree for my report to be made available under an Open Access Creative Commons CC-BY license (http://creativecommons.org/licenses/by/4.0/). I understand that any comments which I do not wish to be included in my named report can be included as confidential comments to the editors, which will not be published.

Choose an item.

To further support our reviewers, we have joined with Publons, where you can gain additional credit to further highlight your hard work (see: https://publons.com/journal/530/gigascience). On publication of this paper, your review will be automatically added to Publons, you can then choose whether or not to claim your Publons credit. I understand this statement.

Yes Choose an item.
