# Supplementary material for: MIFE and MIFD: Minimum information for fermentation experiments and devices
Source: Gigascience. 2026 Apr 9;15:giag038. doi: 10.1093/gigascience/giag038 (PMC13184968; doi:10.1093/gigascience/giag038)
Supplement: giag038_Reviewer_2_Report_original_submission — Reviewer 2 -- 9/4/2025 [file giag038_reviewer_2_report_original_submission.pdf]

Reviewer Report

Title: MIFE and MIFD: Minimum information for fermentation experiments and devices

Version: Original Submission **Date:** 9/4/2025

Reviewer name: Reviewer 2

Reviewer Comments to Author:

The manuscript describes the development of two new standards, minimum information for fermentation experiments (MIFE) and devices (MIFD), addressing challenges in these communities to report data within a standardized framework.
Well written, sufficient justification provided in the background section
-- when providing examples, remove 'etc
-- Overview of existing minimum information models
In this section, I would have expected consideration of OBI: Ontology for Biomedical Investigations.
Are there portions of OBI that would be pertinent to the developed standards?
-- Briefly outline methods utilized to identify/map individual terms within the standards to other standards,
and how these mappings/re-use of terms were integrated.
It is noted in the Aims and Objective section:
Some metadata terms included in MIFD are mapped to existing ontologies.
-- How many terms, from which ontologies?
What version of each ontology were the terms mapped to?
--In the Design Considerations section, it is noted:
-- "what ontological class from an existing ontology it extends"
and
"the URL of a matching, existing, ontology concept ("url" column"
--> Is the ID of the original term retained ?
- Two edits for this sentence:
from:
Minimum Information about any (X) Sequence (MIXS) and its subcategories from the Genomics Standards Consortium
to:
Minimum Information about any (X) Sequence (MIxS) and its subcategories from the Genomic Standards Consortium
- Include the specific license associated with the standards.e.g., CCBY or CCO
-- Add this to the Availability statement.
-- when using e.g. -- always add a comma at the end, e.g.,
-- At the end of the Ontology and FAIR Data Station, there is a e.g. without the comma
-- Figure 4 - the text in the figure is blurry and the font size could be increased for readability
-- Conclusion

-- Is there a mention in the manuscript for gathering and integrating updates to the standards?
How is this planned for ?
Do you want users to submit issues to:
https://gitlab.com/bioindustry-4.0/mim_ontology
-- Are there datasets in preparation for this standard?
-- Where will the datasets be submitted ?
-- How often will updates to the schmema be made available, that is, what is the planned update cycle?

Methods

Are the methods appropriate to the aims of the study, are they well described, and are necessary controls included? Choose an item.

Conclusions

Are the conclusions adequately supported by the data shown? Choose an item.

Reporting Standards

Does the manuscript adhere to the journal's guidelines on minimum standards of reporting? Choose an item.

Choose an item.

Statistics

Are you able to assess all statistics in the manuscript, including the appropriateness of statistical tests used? Choose an item.

Quality of Written English

Please indicate the quality of language in the manuscript: Choose an item.

Declaration of Competing Interests

Please complete a declaration of competing interests, considering the following questions:

- Have you in the past five years received reimbursements, fees, funding, or salary from an organisation that may in any way gain or lose financially from the publication of this manuscript, either now or in the future?
- Do you hold any stocks or shares in an organisation that may in any way gain or lose financially from the publication of this manuscript, either now or in the future?
- Do you hold or are you currently applying for any patents relating to the content of the manuscript?

- Have you received reimbursements, fees, funding, or salary from an organization that holds or has applied for patents relating to the content of the manuscript?
- Do you have any other financial competing interests?
- Do you have any non-financial competing interests in relation to this paper?

If you can answer no to all of the above, write 'I declare that I have no competing interests' below. If your reply is yes to any, please give details below.

I declare that I have no competing interests.

I agree to the open peer review policy of the journal. I understand that my name will be included on my report to the authors and, if the manuscript is accepted for publication, my named report including any attachments I upload will be posted on the website along with the authors' responses. I agree for my report to be made available under an Open Access Creative Commons CC-BY license (http://creativecommons.org/licenses/by/4.0/). I understand that any comments which I do not wish to be included in my named report can be included as confidential comments to the editors, which will not be published.

Choose an item.

To further support our reviewers, we have joined with Publons, where you can gain additional credit to further highlight your hard work (see: https://publons.com/journal/530/gigascience). On publication of this paper, your review will be automatically added to Publons, you can then choose whether or not to claim your Publons credit. I understand this statement.

Yes Choose an item.
